# Dietary resilience of coral reef fishes to habitat degradation

**DOI:** 10.1111/1365-2656.70196

**Published:** 2025-12-14

**Authors:** Friederike Clever, Richard F. Preziosi, Bryan Nguyen, Brígida De Gracia, Helio Quintero Arrieta, W. Owen McMillan, Andrew H. Altieri, Aaron O'Dea, Nancy Knowlton, Matthieu Leray

**Affiliations:** ^1^ Smithsonian Tropical Research Institute Panama Republic of Panama; ^2^ Department of Natural Sciences Manchester Metropolitan University Manchester UK; ^3^ School of Biological and Marine Sciences University of Plymouth Plymouth UK; ^4^ Center for Biomolecular Science and Engineering, U.S. Naval Research Laboratory Washington District of Columbia USA; ^5^ Department of Environmental Engineering Sciences University of Florida Gainesville Florida USA; ^6^ Sistema Nacional de Investigación, SENACYT Panama Republic of Panama; ^7^ National Museum of Natural History Smithsonian Institution Washington District of Columbia USA; ^8^ School of Biological Sciences Swire Institute of Marine Science, The University of Hong Kong Hong Kong SAR China

**Keywords:** *Chaetodon*, COI, corallivore, dietary metabarcoding, *Hypoplectrus*, mesopredator, otolith analysis, trophic interaction

## Abstract

1. The ability of consumers to adjust their diet in response to resource shifts is a key mechanism allowing the persistence of populations and underlying species' adaptive capacity. Yet on coral reefs, one of the marine habitats most vulnerable to global change, the extent to which species alter their diet and the consequences of dietary shifts for consumer performance and ecosystem functioning remain poorly understood.
2. Here, we tested how dietary versatility can mediate the effects of habitat degradation on two invertivorous reef fishes—*Chaetodon capistratus*, a browser, and *Hypoplectrus puella*, an active predator—and whether diet shifts relate to variation in body condition and growth.
3. We integrated DNA‐based gut content analyses (metabarcoding), otolith analysis, body condition and field surveys to link diet profiles to growth and relative body condition across reefs differing in coral cover.
4. Metabarcoding revealed significant dietary variation in both species across reefs with different levels of coral cover. However, the response was more pronounced in the browser, whose diet was anthozoan‐dominated on healthier reefs, whereas it was annelid‐dominated on degraded reefs. We found significantly more variable body condition on degraded reefs in the browser, while the body condition of the active predator decreased in larger individuals on degraded reefs.
5. Our results suggest that while dietary versatility serves as a mechanism to cope with degraded environments, the degree to which dietary shifts can buffer against the effects of habitat degradation varies between species. Overall, the variation in trophic niche across sites suggests that food webs and energy flow differ at relatively small scales between healthy and degraded reefs.

## Graphical abstract

Metabarcoding of gut contents shows that two common benthic‐feeding reef fishes with different feeding stratgies—a butterflyfish (*Chaetodon capistratus*) and a hamlet (*Hypoplectrus puella*)—shift diets on degraded reefs. These shifts mirror contrasting patterns in body condition: butterflyfish showed strong individual variation, whereas condition was only reduced in larger hamlets on degraded reefs, suggesting species‐specific coping mechanisms in response to coral loss.

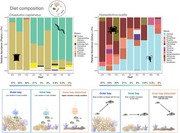

## Resumen

1. La capacidad de los consumidores para ajustar su dieta en respuesta a los cambios en la disponibilidad de recursos es un mecanismo clave que permite la persistencia de las poblaciones y la capacidad de adaptación de las especies subyacentes. Sin embargo, en los arrecifes de coral, uno de los hábitats marinos más vulnerables al cambio global, aún se desconoce en gran medida hasta qué punto las especies alteran su dieta y las consecuencias de los cambios alimentarios para el rendimiento de los consumidores y el funcionamiento del ecosistema.
2. En este estudio, probamos cómo la versatilidad alimentaria puede mediar los efectos de la degradación del hábitat en dos peces invertívoros de arrecife: *Chaetodon capistratus*, un ramoneador, e *Hypoplectrus puella*, un depredador activo, y si los cambios en la dieta se relacionan con la variación en la condición corporal y el crecimiento.
3. Integramos análisis del contenido intestinal basados en ADN (metabarcoding), análisis de otolitos, condición corporal y censos visuales para relacionar la composición de la dieta con el crecimiento y la condición corporal relativa de peces en arrecifes con diferente cobertura de coral.
4. El metabarcoding reveló una variación significativa en la dieta de ambas especies a lo largo de arrecifes con diferentes niveles de cobertura de coral. Sin embargo, la respuesta fue más pronunciada en el ramoneador, cuya dieta estaba dominada por antozoos en arrecifes más sanos, mientras que en arrecifes degradados estuvo dominada por anélidos. Encontramos que la condición corporal es significativamente más variable en arrecifes degradados en el ramoneador, mientras que la condición corporal del depredador activo disminuyó en los individuos más grandes en arrecifes degradados.
5. Nuestros resultados sugieren que, si bien la versatilidad alimentaria sirve como mecanismo para hacer frente a los entornos degradados, el grado en que los cambios alimentarios pueden amortiguar los efectos de la degradación del hábitat varía entre las especies. En general, la variación en el nicho trófico entre los distintos sitios sugiere que las redes tróficas y el flujo de energía difieren a escalas relativamente pequeñas entre los arrecifes sanos y los degradados.

## 1 INTRODUCTION

The capacity to take advantage of alternative resources is increasingly recognized as an important trait for species' resilience in the face of habitat degradation (MacNally, 1995; Wong & Candolin, 2015). Such changes in feeding behaviour, in turn, influence community dynamics (e.g. mediated by relative levels of niche overlap among species) and trophic interactions, which underpin ecosystem functioning. Despite implications for both population persistence and ecosystem functioning, little is known about dietary versatility in the wild in response to changing resource landscapes and the potential consequences for consumers. This is particularly true in marine environments, where empirical studies of species dietary niches are challenging due to the complexity of marine food webs and the difficulty of documenting trophic behaviours (Donelson et al., 2019). However, with evolving high‐throughput sequencing technology it is now possible to analyse large quantities of gut content samples simultaneously, illuminating diets at unprecedented taxonomic resolution and spatial scale (Alberdi et al., 2019; Pompanon et al., 2012).

On coral reefs, fishes are subjected to accelerating rates of severe habitat change and degradation. This has led to declines in abundance across most trophic groups (Pratchett et al., 2018). Specialized species, such as obligate corallivores, are thought to be the most vulnerable (Graham, 2007; Wilson et al., 2006). Generalized feeding strategies, as observed in some species of coral reef fishes, may be more resilient to changes in resource availability (Wilson et al., 2006), but to which degree trophic versatility provides population resilience to habitat degradation remains poorly understood. This is in part because alternative prey choice may entail lowered nutritional uptake and thus reduce fish health condition with potentially negative consequences for fitness and population persistence (Berumen et al., 2005; Hempson et al., 2017; Pratchett et al., 2004). However, describing complex consumer–resource interactions has been hampered by limited empirical data at adequate prey taxonomic resolution for detecting potentially subtle dietary variation (Parravicini et al., 2020), especially in generalist feeders with flexible diets. As a consequence, more detailed knowledge of dietary resource use is required to understand responses to habitat and prey community change in coral reef fishes.

Here we leverage dietary metabarcoding (i.e. the DNA‐based characterization of prey communities in stomachs and guts) to assess levels of dietary versatility of two common coral reef fishes across reefs that vary markedly in coral cover in a Caribbean system. Fish diet information derived from gut contents has been commonly studied visually by describing the morphological features and hard part remains of prey organisms (Baker et al., 2014; Nielsen et al., 2017; Randall, 1967; Traugott et al., 2020), and/or with behavioural observations of foraging and bite rates (Baker et al., 2014; Hyslop, 1980; Pratchett, 2005). The main advantage of dietary metabarcoding over these conventional visual approaches is that it allows identification of semi‐digested, soft‐bodied, small and/or cryptic organisms (meiofauna and microbiota) (Chariton et al., 2015; Leray & Knowlton, 2015) that may not be detected visually (Berry et al., 2015; Nagelkerken et al., 2009). The increased taxonomic resolution offered by DNA metabarcoding has already revealed higher levels of resource partitioning among closely related species than previously assumed (Brandl et al., 2020; Leray et al., 2015, 2019) and, conversely, unexpected patterns of dietary overlap (Coker et al., 2022). By enabling prey identification to an unprecedented taxonomic resolution, DNA metabarcoding allows detection of intraspecific variations in diet that would otherwise go unnoticed, and this in turn can be linked to changes in consumer populations and prey availability to understand the responses to habitat degradation.

We took advantage of DNA metabarcoding to explore the dietary patterns of two common reef fish species with distinct feeding strategies: the browsing butterflyfish *Chaetodon capistratus* and the hamlet *Hypoplectrus puella*, an active predator. Our study encompasses nine reefs that vary in coral cover and benthic composition situated on the Caribbean coast of Panama. We quantified links between diet (composition and breadth), fish age, growth and body condition and prey densities across reefs of extremely low (~0.3%) to intermediate (~13%) to relatively high levels (~30%) of coral cover, created by severe hypoxic events inside Bahía Almirante in Bocas del Toro, Panama, providing conditions of a natural experiment (Altieri et al., 2017; Leray et al., 2021). Based on the literature (Birkeland & Neudecker, 1981; Gore, 1984) and on feeding observations (F.C. unpublished data), we hypothesized that *C. capistratus* would switch from an anthozoan‐dominated diet on high coral cover reefs to a broader suite of prey taxa on reefs where coral cover is very low. In contrast, we expected *H. puella* to maintain a high proportion of crustaceans in its diet across all reefs (e.g. Whiteman et al., 2007), but with compositional variation as a function of change in benthic invertebrate assemblages in relation to coral cover.

## 2 MATERIALS AND METHODS

### 2.1 Study system

**FIGURE 1:**
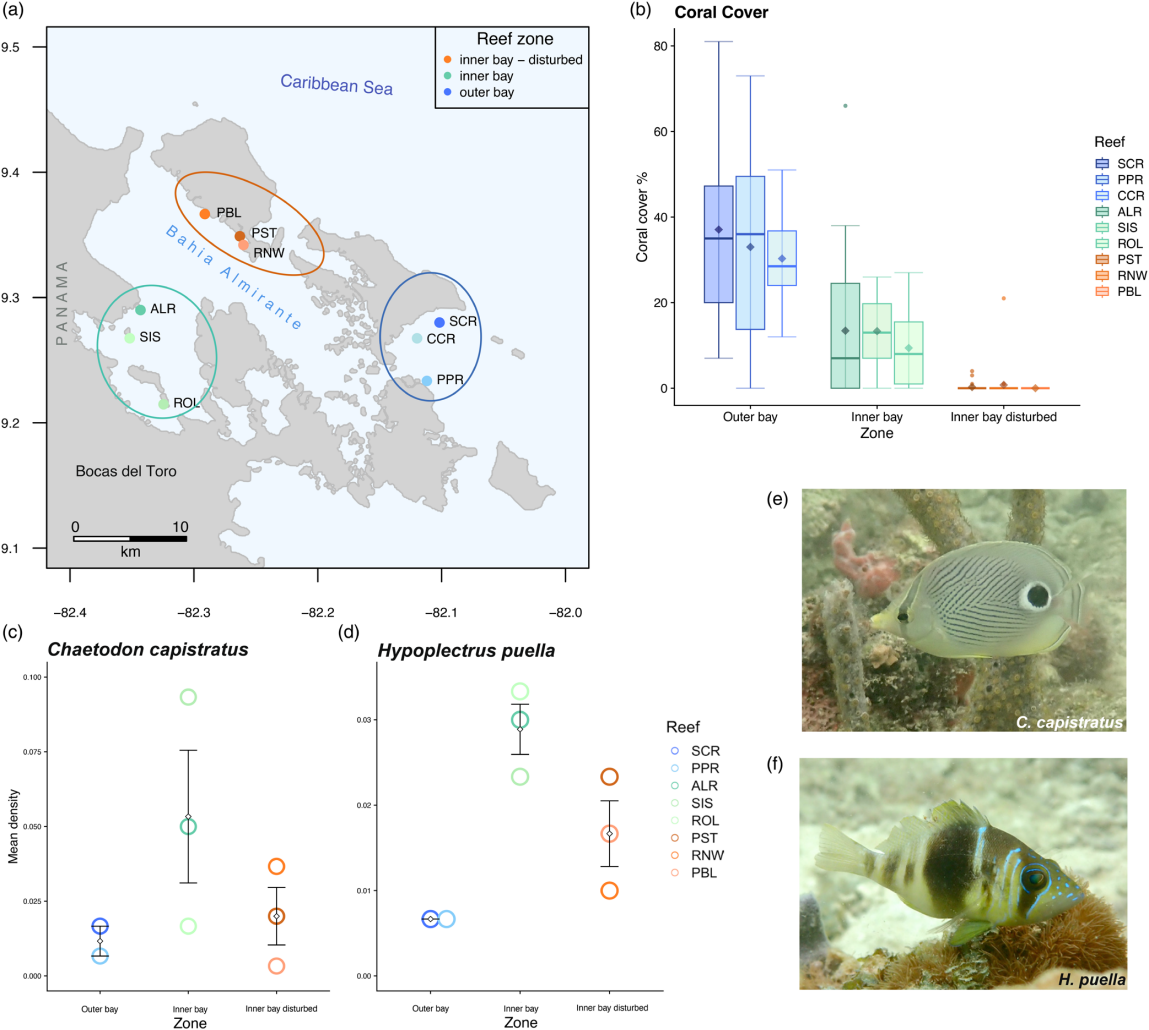
Study system in Bahía Almirante, Bocas del Toro (Panama). (a) Map locating the nine study reefs across three zones characterized by different levels of live coral cover: Outer bay reefs (blue) [Salt Creek (SCR), Cayo Corales (CCR) and Popa (PPR)]; inner bay reefs (green) [Almirante (ALR), Cayo Hermanas (SIS) and Cayo Roldan (ROL)]; inner bay disturbed reefs (orange) [Punta Puebla (PBL), Punta STRI (PST) and Runway (RNW)]. (b) Percent live hard coral cover across the habitat zones from high coral cover (outer bay zone) to low coral cover (inner bay disturbed zone), boxplot upper and lower whiskers correspond to the first and third quartiles, bars depict medians, diamonds depict means. Boxplots are each based on data from 10 quadrats per transect with 3 transects per reef (*N* = 30 quadrats per reef). Fish density (mean ± SD) across reef zones for (c) *Chaetodon capistratus* and (d) *Hypoplectrus puella*. (e) *Chaetodon capistratus* and (f) *Hypoplectrus puella*. Photos: Matthieu Leray.

Bahía Almirante is a large (450 km^2^), semi‐enclosed coastal lagoon in the Bocas del Toro Archipelago on the Caribbean coast of Panama (Figure 1a). Its specific geomorphology, climate and human pressures contribute to occasional reductions in dissolved oxygen levels. The bay is confined by the mainland and protected from ocean swell by several islands leading to restricted water exchange with the open ocean. Together with local climatic conditions, this creates an environment with limited water flow, variable salinity (fluctuating locally from 20 to 34 PSS) (Collin et al., 2009; Kaufmann & Thompson, 2005) and elevated sea‐surface temperatures during calm weather periods (Altieri et al., 2017; Cramer, 2013). While terrestrial run‐off naturally elevates nutrients within the bay, both untreated wastewater from tourism development and agricultural discharges intensify eutrophic levels (Altieri et al., 2017; Cramer, 2013; D'Croz et al., 2005; Guzmán et al., 2005). In 2010, a hypoxic stress event led to drastic coral cover decline and die‐off (Altieri et al., 2017) that contributed to a gradient of habitat degradation across the bay as coral cover sharply decreased on reefs in areas inside the bay (Figure 1a, ‘inner bay disturbed’ zone), but less so or not at all in other areas of the inner bay (Figure 1a, ‘inner bay’ zone) and outside the bay (Figure 1a, ‘outer bay’ zone; Figure 1b). We tested how habitat affects the diet and body condition of two species of reef‐associated, benthic‐feeding fishes across these zones (Figure 1c–f). We selected three discrete reefs from each of three reef zones based on coral cover data: ‘outer bay’ (high coral cover), ‘inner bay’ (intermediate coral cover) and ‘inner bay disturbed’ (very low coral cover) (*N* = 9 reefs total, Figure 1a,b). Other related factors, such as water quality and exposure likely covary with coral cover across the area and may contribute to relative prey availability (Collin et al., 2009; D'Croz et al., 2005; Kaufmann & Thompson, 2005).

### 2.2 Study species

Both study species are coral‐associated, benthic carnivores that are common members of Caribbean reef fish assemblages (‘least concern’ conservation status, IUCN; Anderson et al., 2015; Rocha et al., 2010; Figure 1e,f). The barred hamlet *Hypoplectrus puella* (Cuvier) is a small sea bass (Perciformes: Serranidae) known to prey mainly upon crustaceans and to a lesser extent upon fishes (Holt et al., 2008; Randall, 1967; Whiteman et al., 2007) within small foraging territories, often in sit‐and‐wait mode (Barlow, 1975). The foureye butterflyfish *Chaetodon capistratus* (Linnaeus) feeds primarily by browsing on anthozoans with a preference for scleractinian corals (Birkeland & Neudecker, 1981; Liedke et al., 2018). It tends to feed almost continuously while visiting various colonies within a reef zone (e.g. crest), usually for short feeding bouts during which it nips on coral polyps (Pitts, 1991), but distances travelled may vary (Gore, 1983).

### 2.3 Benthic, fish and invertebrate surveys

To characterize habitat quality and fish populations, benthic cover and reef fishes were surveyed in May and June of 2016 at our nine study reefs (research permit No. SE/A‐44‐16, Ministerio de Ambiente Panamá). Coral cover levels remained stable between 2016 and our fish collections in 2018 (Doucette et al., 2022). Using three replicate 20 m transects per reef, we assessed benthic cover from photo quadrats, fish communities and study species' densities along 20 × 5 m belts, and assessed diversity and abundance of macroinvertebrates (>2 mm) within three quadrats per reef (50 × 50 cm). Three replicate transect lines (20 m) per reef were placed parallel to the shore at a depth of 2–4 m. To estimate benthic cover and community composition, ten quadrats (100 × 70 cm) were photographed at 2 m increments along each transect (quadrats per site *N* = 30). We analysed each photo using CoralNet (Beijbom et al., 2015) with a grid of 100 points over each photo, and the identity of the primary space holder under each point was determined to the level of broad taxonomic groups (e.g. hard coral, soft coral, macroalgae, sponge, dead coral, zoanthids, rubble) to avoid potential identification errors arising from variation in image quality. Hard corals were identified at the genus level where possible. Fish communities were surveyed along a 20 × 5 m belt by one experienced surveyor recording the abundance and identity of all non‐cryptic fish species using scuba. To assess macro‐invertebrate (>2 mm) diversity and abundance, quadrats (50 × 50 cm) were placed on continuous surfaces of dead coral (mainly *Agaricia tenuifolia*) and all coral rubble was carefully collected in bins and transported to the field station. At the laboratory, all invertebrates larger than 2 mm were counted and identified to the lowest taxonomic level.

### 2.4 Fish collection

To characterize fish body condition and collect diet material, 20 adult fishes per species were collected at each of the nine reefs by spearfishing (Figure 1a) in February and March of 2018 following protocols approved by the Institutional Animal Care and Use Committee of the Smithsonian Tropical Research Institute (IACUC; Proposal No. 2018‐0125‐2021). A research permit was issued by the Ministerio de Ambiente Panamá (No. SE/A‐113‐17). Immediately after spearing, each fish was anaesthetised on the boat in a sterile and labelled Whirl‐Pak bag with seawater and clove oil and subsequently stored on ice. Upon return to the field station, fish were weighed (wet weight, g) and total length (TL, mm) was measured using a digital calliper. Each fish was dissected under a laminar flow hood using sterile, DNA de‐contaminated tools. Gastrointestinal tracts were individually preserved in 96% ethanol and stored at *−*20*°*C until DNA extraction. Strict procedures were used to avoid cross‐contamination (Supporting Information Methods, section III).

### 2.5 Otolith‐based fish age determination and growth rate estimation

Pairs of sagittal otoliths were extracted from fish individuals (*C. capistratus N* = 158, *H. puella N* = 127) and photographed using a LEICA Model EZ4W stereoscopic microscope with an integrated camera and light system (Figure S1A,B). One otolith of each pair was analysed for annuli following the methods in Morales‐Nin (1991) (Figure S1C). Daily growth increments were examined for a subset of otoliths of each species to confirm that each annulus corresponds to 1 year of growth. Five otoliths (one *C. capistratus* and four *H. puella*) were removed from the study because of anomalous annuli. The total length of each remaining otolith (LO) was measured (*C. capistratus N* = 117, *H. puella N* = 127) while positioning the otoliths on their distal sides.

### 2.6 Prey tissue preparation and DNA extraction

The digestive tract of each fish was separated into stomach and intestine. The stomach content represents a snapshot of the most recent prey ingested, whereas the intestinal content integrates semi‐digested prey consumed up to multiple hours prior to collection. The stomachs of *C. capistratus*, and intestines of *H. puella*, were dissected longitudinally and contents and digesta isolated respectively. The stomachs in *C. capistratus* contained a diverse and representative assortment of prey items, presumably because of the species' continuous browsing behaviour. In contrast, the stomachs of the sit‐and‐wait occasional predator *H. puella* frequently contained only a single prey item, and we therefore sampled intestines that yielded more diverse representations of prey items. Prey tissue was removed from stomachs and digesta and mucosa isolated from intestines using sterile and DNA‐decontaminated forceps and disposable sterile surgical blades. Gut mucosa was included here as samples were also used for analysis of bacterial communities (not presented in this study) (Clever et al., 2022). Isolated stomach and intestinal contents were then weighed (wet weight mg) individually on clean, sterile weighing boats on a digital scale. Dissection and extraction blanks were introduced at this step by performing each preparation step with a sample consisting of nuclease‐free water. One negative control was included in each set of extractions (~20 samples). DNA was extracted from between 0.05 and 0.25 g of prey tissue per sample using the Qiagen Powersoil DNA isolation kit following the manufacturer's instructions with minor modifications to increase the yield (Supporting Information Methods, section II). DNA was eluted in 100 μl buffer (C6 solution).

### 2.7 Metabarcoding library preparation

To enable identification of prey items to the species level, we targeted a 313 bp fragment of the hyper variable mitochondrial Cytochrome c Oxidase subunit I (mtCOI) gene region with a versatile PCR primer set (mlCOIintF and jgHCO2198; Geller et al., 2013; Leray, Yang, et al., 2013; Table S1). This primer set, originally designed for the amplification of metazoan DNA, was shown to be effective at characterizing coral reef fish gut contents (Leray, Yang, et al., 2013) and has previously successfully amplified diverse bulk samples of marine benthic taxa as well as provided useful abundance estimates (Leray & Knowlton, 2015) despite known methodological biases (Pompanon et al., 2012). Therefore, the use of this primer set allowed us to leverage read counts as a coarse measure of prey proportions in our downstream analyses, rather than relying solely on occurrence data which would have equally weighted rare and common prey items in diets (Deagle et al., 2019). In each PCR reaction, we included consumer‐specific annealing blocking primers (Table S2) (at 10× COI primers), as amplification of consumer DNA can overwhelm the recovery of prey (Vestheim & Jarman, 2008). Blocking primer design and thermocycling parameters followed the methods described in Leray, Agudelo, et al. (2013). Polymerase Chain Reaction (PCR) was carried out for three replicates of each sample to enhance prey detection probability and account for variation in PCR amplifications caused by PCR drift (Alberdi et al., 2017; De Barba et al., 2014). We employed a PCR‐free library preparation approach with matching tags using the TruSeq DNA PCR‐free LT library Prep Kit (Illumina). Our methods for sample multiplexing, PCR reactions and library preparation are detailed in the Supporting Information Methods (section V). Sequencing of the final product was performed on an Illumina MiSeq sequencer (reagent kit version 2, 500 cycles) at the George Washington University, Washington, DC.

### 2.8 Sequence analysis

Bioinformatics steps were performed in R (v3.6.1; R Core Team, 2019). After demultiplexing, sequence reads were adapter‐, primer‐ and quality‐trimmed with Flexbar (v3.0.3; Roehr et al., 2017). Subsequently, sequences were filtered, chimera‐checked, and processed into amplicon sequence variants (ASVs) with DADA2 (v1.9.0; Callahan et al., 2016). ASVs were then clustered with VSEARCH (Rognes et al., 2016) at a 97% identity threshold into OTUs to approximate biological species. OTUs were curated with the LULU algorithm (Frøslev et al., 2017) by reducing taxonomic redundancy and enhancing the richness estimate accuracy (LULU parameters: minimum ratio type = ‘min’, minimum ratio = 1, minimum match = 84, minimum relative co‐occurrence = 0.95). OTUs were assigned taxonomy using the Bayesian Least Common Ancestor (BLCA) taxonomic classifier (Gao et al., 2017) against the Midori‐Unique v20180221 database (Leray et al., 2022; Machida et al., 2017), which is a curated library of metazoan COI sequences (available at www.reference‐midori.info). We omitted all BLCA taxonomy assignments of less than 50% confidence. Unassigned OTUs were identified using BLAST searches (word size = 7; max e‐value = 5e‐13) against the whole NCBI NT database (retrieved May 2018), and the lowest common ancestor of the top 100 hits was used to assign taxonomy. We retained all OTUs delineated as Metazoa for downstream statistical analysis. All OTUs delineated as either one of our fish study species were removed.

### 2.9 Statistical analyses

#### 2.9.1 Benthic, fish and invertebrate surveys

To determine the mean percent coral cover at each reef, we first calculated the mean counts (hard coral) across photo quadrats on each transect, and then took the mean across all transects (*N* = 3) at each reef. We then estimated Shannon diversity (*H*′) for hard corals at each reef. Significant differences in mean percent coral cover and coral diversity among zones were assessed using the Kruskal–Wallis test (Kruskal & Wallis, 1952) with Benjamini–Hochberg corrected post hoc Dunn's test (Benjamini & Hochberg, 1995; Dunn, 1964). Differences in benthic composition among three zones were visualized using principal coordinates analysis (PCoA) based on Bray–Curtis dissimilarity (Bray & Curtis, 1957). Non‐metric multidimensional scaling (NMDS; Clarke & Warwick, 2001) with Bray–Curtis dissimilarity was used to assess differences in fish communities among habitat zones. To estimate mean densities of the study fish species at each reef, the total number of fish was divided by the transect area and the mean and standard deviation across the three replicate transects were calculated. We visualized mean densities across zones with dot plots and tested for significant differences among zones using one‐way analysis of variance (ANOVA) with post hoc Tukey test. To analyse variation in the density of mobile invertebrates frequently consumed by our target fish based on metabarcoding results, we calculated the density of each taxon (e.g. arthropods) for each quadrat (sum divided by quadrat area) and calculated mean densities across quadrats (*N* = 3) on each reef. Variation in mean densities was compared at various taxonomic levels (i.e. all sampled invertebrates, arthropods, decapods, true crabs [Brachyura], mithracid crabs, spaghetti worms [Terebellidae]) among zones using boxplots and significant differences assessed by ANOVA with post hoc Tukey test. Both overall invertebrate and arthropod community composition were visualized using stacked barcharts of relative densities.

#### 2.9.2 Fish length–weight and body condition

Total length (TL) and wet weight (W) mean ± SD were calculated for both species and Kruskal–Wallis tests with Benjamini–Hochberg corrected post hoc Dunn's tests were used to assess differences among zones with the exception of weight (W) for *H. puella* where an ANOVA with post hoc Tukey test was used because the data were normally distributed. We then modelled the length–weight relationship by species for the whole dataset using linear regressions

logW∽logL

where *L* and *W* are the natural log transformed fish total length (mm) and wet weight (g), respectively. To compare fish body condition (e.g. relative ‘plumpness’ in relation to length—with plumper fish of a given length assumed to be in better condition; Froese, 2006; Tesch, 1968) among zones, we calculated the relative condition factor (*K*_n_) (Le Cren, 1951) for each individual by estimating the deviation between the observed weight to the predicted length‐specific mean weight of the population (Blackwell et al., 2000; Froese, 2006)

$$K_{n}=W:aL^{b^{\prime }}$$

where *a* and *b* are the species‐ and population‐specific length–weight parameters, respectively, obtained from the length–weight regression. *L* is the natural log transformed observed total length (mm) and *W* is the natural log transformed observed weight (g). Due to the local scope of our study focusing on small‐scale spatial differences among fish subpopulations within species, the relative condition factor (*K*_n_) was used as opposed to the relative weight (*wr*), the latter being based on standard weight developed across populations (Blackwell et al., 2000). To assess how fish condition varied across zones, we first tested if relative condition (*K*_n_) varied among zones overall using Kruskal–Wallis tests, with effect sizes calculated as epsilon squared (*ε* ^2^). Second, we plotted relative fish condition *K*_n_ against fish total length (cm) for each zone, respectively. Lastly, we fitted condition–length regression models to assess whether the slopes differed among zones. We modelled the interaction between fish total length and zone in affecting the condition–length relationship

$$K_{n}∼\log L*\text{Zone}$$

ANOVA was used to determine whether slopes differed significantly. Model predictions with confidence intervals were generated (transformed data with bias correction) and visualized as scatterplots. Effect sizes (partial *η* ^2^) were calculated from Type I ANOVA. Pairwise comparisons of interaction slopes were performed by estimating marginal trends with Tukey‐adjusted *p*‐values. Log variance ratios (lnVR) (Nakagawa et al., 2015) on model residuals (*K*_n_ ~ log*L* * Zone) and residual plots were used to examine if variation in *K*_n_ differed significantly among zones while controlling for fish size, as the magnitude of spread may reflect divergent individual responses to local conditions. Pairwise log variance ratios (lnVR) were calculated with confidence intervals generated through bootstrapping (1000 resamples) and statistical significance was inferred where intervals did not overlap zero. In addition, we tested for significant differences in wet weight (g) and total length (mm) among zones (ANOVA).

#### 2.9.3 Age and growth

We tested for significant differences in fish age among zones based on otolith analyses (Kruskal–Wallis test and post hoc Dunn's test with Benjamini–Hochberg correction). Then we fitted von Bertalanffy growth functions (VBGF)

$$L\text{(}\text{t}\text{)}={\text{L}}_{\text{∞}}\;\left(1-e^{-K\;\left(t-t_{0}\right)}\right)$$

where *L*(*t*) is the length at age *t*, *L*_∞_ (length infinitive) is the asymptotic length, *K* is the growth rate at which asymptotic length is approached and *t*_0_ is the hypothetical age (years) at which length is zero. To do so, we modified R functions from the FSA package (v 0.9.1; Ogle et al., 2020) and plotted curves visualizing model prediction values. Bootstrapped predictions were used to test for significant differences in asymptotic length (*L_∞_*) and growth rate (*K*) among the three zones for each species.

#### 2.9.4 Diet composition and specialization

NMDS ordinations (Clarke & Warwick, 2001) based on Bray–Curtis dissimilarity were used to visualize differences in diet composition among reefs and zones for each fish species. We then tested for significant differences in diet composition among three zones using permutational multivariate analysis of variance (PERMANOVA) with 10,000 permutations. The pairwiseAdonis function was computed with Bonferroni‐corrected *p*‐values for pairwise comparisons between zones (Martínez Arbizu, 2019). Stacked barcharts were generated for each fish species depicting relative read abundances of prey OTUs across nine study reefs. We then asked if percent coral cover and/or fish age significantly affected the sequencing read abundance of main diet items as identified by metabarcoding: (i) annelids and hard corals in *C. capistratus*, and (ii) benthic and planktonic crustaceans in *H. puella*, fitting separate generalized linear mixed models (GLMMs) with a negative binomial distribution for each combination of response and predictor. We accounted for the hierarchical structure of our study design by including random effects of zone with reef nested within zone in GLMMs. For the hard coral diet item, we included random effects of zone only because the availability of hard coral prey is strongly collinear with coral cover estimates. For non‐coral diet items, we used the nested random effects (zone/reef) as other aspects of reef habitat could still affect their availability. The distribution of data was checked using histograms and transformed to optimize models, that is, hard coral was square root‐, annelid fourth root‐ and both crustaceans and fish log transformed. Likelihood ratio tests were performed to test the significance of the predictor variables against null models (e.g. models with and without specific predictors). Data were visualized using *q* plots and model fit was examined with Q‐Q plots. To characterize the feeding strategy of both fish species in terms of how specialized or generalized the diet appears on the population level, we used a graphical analysis proposed by Amundsen et al. (1996) modified from Costello (1990). To generate diagrams representing feeding strategy and prey importance at three reef zones, frequency of occurrence was expressed as a percentage and calculated by dividing the number of fish individuals in which a prey item was present by the total number of fish. Prey‐specific abundance was calculated as the percentage of the diet that a food item represents across only those fish individuals where it was present

$$P_{i}=\left(\;\sum S_{i}⁄\;\sum {\mathrm{St}}_{i}\;\right)*100$$

where *P*_*i*_ is the prey‐specific abundance of prey *i*, *S*_*i*_ is the abundance of prey *i* in the stomach or intestines, and *S*_*ti*_ is the total prey abundance in only consumer individuals where prey *i* is present. Because a generalist diet profile may arise at the population level from either broad individual diets and/or high variation in diet composition among specialized individuals (Amundsen et al., 1996; Bolnick et al., 2003), Amundsen's method includes an indirect measure of the contribution to niche width of both within individual variation (within phenotype component, WPC) and variation among individuals (between phenotype component, BPC).

All statistical analyses were performed in R (v4.4.2; R Core Team, 2024). Significance testing used kruskal.test, aov, and TukeyHSD (stats v 4.1.3). Permutational ANOVA was performed with adonis2 (vegan v2.6‐10; Oksanen et al., 2025). Statistical modelling used glmer.nb (lme4 v1.1‐21; Bates et al., 2015), lm, anova (stats), and dunnTest (FSA v0.8.30; Ogle et al., 2020). Pairwise estimated marginal trends were obtained with emtrends (emmeans v1.11.1; Lenth et al., 2025). Effect sizes and supporting packages were computed with effectsize (v1.0.0; Ben‐Shachar et al., 2025), and rcompanion (v2.5.0; Mangiafico, 2025). Predicted values were generated with ggpredict (ggeffects v2.2.0). Log variance ratios (lnVR) were calculated using purrr (v1.0.4; Wickham & Henry, 2025), boot (v1.3‐31; Canty & Ripley, 2024) and dplyr (v1.1.4; Wickham et al., 2025) packages. Ordinations were generated with ordinate (phyloseq v1.50.0; McMurdie & Holmes, 2013), metaMDS (vegan v2.6‐10; Oksanen et al., 2025), and cmdscale (stats v4.4.2; R Core Team, 2024).

## 3 RESULTS

### 3.1 Benthic, fish and invertebrate surveys

The outer bay zone featured the highest levels of live coral cover (percent cover per transect across three reefs mean ± SD = 33.46 ± 3.41, Figure 1b) and coral diversity (Shannon diversity, Figure S2). Live coral cover and coral diversity were lower at the inner bay zone (12.06 ± 2.3; Figure 1b, Figure S2), and live corals were nearly absent at reefs in the inner bay disturbed zone (0.34 ± 0.39; Figure 1b), which also had the lowest levels of coral diversity (Figure S2). Percent live coral cover significantly differed among the three reef zones (Kruskal; *χ* ^2^ = 187.01, *p* < 0.001; Figure 1b) and between reef zone pairs (Dunn's adjusted *p*; inner bay–inner bay disturbed: *p* < 0.001; inner bay–outer bay: *p* < 0.001; outer bay–inner bay disturbed: *p* < 0.001). Zones also significantly differed in terms of coral diversity (Shannon diversity) (Kruskal; *χ* ^2^ = 18.13, *p* < 0.001), whereby two of three pairwise comparisons significantly differed (Dunn's adjusted *p*; inner bay–inner bay disturbed: 0.64; inner bay–outer bay: *p* = 0.001; outer bay–inner bay disturbed: *p* < 0.001) (Figure S2). Principal coordinates analysis (PCoA) of benthic composition revealed that differences among zones were driven by sponges and dead corals (inner bay disturbed zone), ‘other invertebrates’ (inner bay zone) and live hard and soft corals with macroalgae (outer bay zone) (Figure S3). Mean fish density peaked at the inner bay zone for both species, but differed significantly only in the case of *H. puella* (ANOVA; *C. capistratus*: *F* = 1.8, *p* = 0.25; *H. puella*: *F* = 10.9, *p* = 0.01) between the outer bay and inner bay zones (Tukey HSD; difference = −0.02, 95% CI: −0.04 to −0.01, *p* = 0.014) (Figure 1c,d). NMDS ordination of fish communities showed clustering of reefs located at the outer bay zone and inner bay disturbed zone, respectively, whereas the inner bay zone was more variable (Figure S4). Overall benthic invertebrate mean density significantly differed among three zones with the highest levels at the outer bay zone (Figure S5A, Table S3); post hoc testing confirmed a significant difference between the inner bay disturbed zone and the outer bay zone (Figure S5A, Table S4). Regarding individual invertebrate groups constituting important fish prey, we found no significant differences in the mean densities of either spaghetti worms (family: Terebellidae, phylum: Annelida) (Figure S5B, Table S3) or crustaceans (phylum: Arthropoda) (Figure S5C, Table S3). Within Arthropoda, there were also no significant differences among zones for decapod crustaceans (order: Decapoda) (Figure S5D, Table S3), Brachyura (true crabs, order: Decapoda) (Figure S5E, Table S3), and mithracid crabs (family: Mithracidae) (Figure S5F, Table S3). Crustaceans of class Malacostraca (phylum: Arthropoda) were more abundant at the inner bay disturbed zone than the outer bay zone (Figure S6A). Within arthropods, the relative densities of decapod crustaceans (class: Malacostraca) were highest at the inner bay zone and at one site (PPR, Popa Reef) of the outer bay, but lower at reefs of the inner bay disturbed zone and Salt Creek Reef (SCR) at the outer bay (Figure S6B).

### 3.2 Fish length–weight relationship and body condition

**FIGURE 2:**
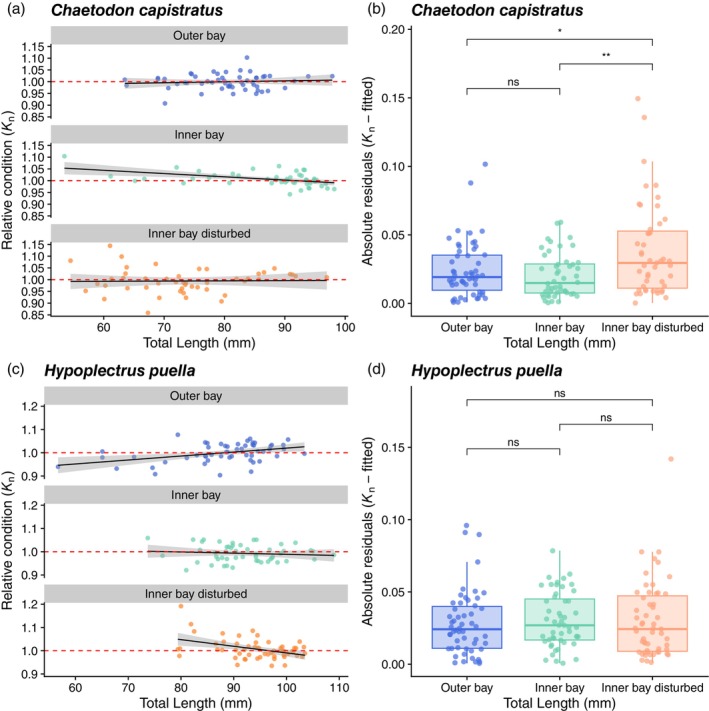
Relative condition (*K*_n_) by fish total length (mm) at three reef zones. Regression lines with 95% confidence intervals depicted in grey show variation in the slopes (*b*) among three reef zones for (a) *Chaetodon capistratus* and (c) *Hypoplectrus puella*. The red dashed line depicts *K*_n_ = 1. Values ≥1 indicate that fish individuals are in good physical condition, values <1 suggest suboptimal condition (a and c). Boxplots of residual spread from the condition‐by‐length model, reflecting differences in the standard deviation of individual condition among zones for (b) *Chaetodon capistratus* and (d) *Hypoplectrus puella*. Significant differences are based on comparing log variance ratios (lnVR) with confidence intervals between zones. Asteriks denote comparisons for which the 95% CI of InVR does not overlap 0 (evidence of a difference); ns indicates the 95% CI includes 0 (no evidence of a difference).

Total length (TL) ranged from 53.49 to 98.19 mm (mean ± SD = 80.08 ± 10.73) for *C. capistratus*, and 56.73 to 125.23 mm (91.19 ± 8.96) for *H. puella*. Wet weight (W) ranged from 5.34 to 34.40 gr (17.97 ± 7.25) for *C. capistratus* and from 3.01 to 23.49 gr (14.60 ± 3.8) for *H. puella*. We found significant differences in fish length (TL) and wet weight (W) among zones for both *C. capistratus* (Kruskal; TL: *χ* ^2^ = 43.97, *p* < 0.001; W: *χ* ^2^ = 43.66, *p* < 0.001) and *H. puella* (Kruskal; TL: *χ* ^2^ = 15.15, *p* < 0.001; ANOVA; W: *F* = 7.23, *p* < 0.001) (Table S5A). All pairwise comparisons significantly differed for *C. capistratus*, whereas *H. puella* was significantly longer and heavier at the inner bay disturbed than at the outer bay zone (Table S5B). The relative fish condition factor (*K*_n_) was at optimal levels (*K*_n_ ≥ 1) at the outer and inner bay zones, and slightly below optimal (*K*_n_ < 1) at the inner bay disturbed zone for *C. capistratus* (Figure S7A). In contrast, *H. puella* showed optimal *K*_n_ values at the outer bay and inner bay disturbed zones (*K*_n_ ≥ 1), with below optimal levels at the inner bay zone (*K*_n_ < 1) (Figure S7B). However, these differences were not statistically significant (Kruskal; *C. capistratus*: *χ* ^2^ = 2.41, *p* = 0.3, ε^2^ = 0.02; *H. puella*: *χ* ^2^ = 2.36, *p* = 0.31, ε^2^ = 0.01) (Figure S7A,B). We found a marginally significant interaction between fish total length and zone affecting the condition–length relationship in *C. capistratus* (ANOVA; *F* = 2.49, *p* = 0.09) (Figure 2a) with condition decreasing with size at the inner bay (slope = −0.11) and increasing at the outer bay (slope = +0.03). However, this pattern appeared to be driven by a few data points, and the effect was small (*partial η* ^2^ = 0.03). In contrast, the interaction differed significantly for *H. puella* (ANOVA; *F* = 11.84, *p* < 0.001; Figure 2c), whereby condition increased with length at the outer bay (slope = +0.14), steeply declined at the inner bay disturbed (slope = −0.26), and slightly declined at the inner bay zone (slope = −0.05). The effect of the interaction was within the moderate to large range (*partial η* ^2^ = 0.13). Post hoc comparisons of the length (TL)–condition (*K*_n_) slopes between zones were not statistically significant for *C. capistratus* (inner bay vs. inner bay disturbed: *p* = 0.17; inner bay vs. outer bay: *p* = 0.13; inner bay disturbed vs. outer bay: *p* = 0.88). For *H. puella*, there was a significant difference in slopes between the outer bay and the inner bay disturbed zones (*p* < 0.001), but not between the outer bay and inner bay zones (*p* = 0.06) and between the inner bay and the inner bay disturbed zones (*p* = 0.08). Log variance ratios (lnVR) revealed ~4‐fold greater variance in fish condition (*K*_n_) at the inner bay disturbed versus at the inner bay zone (lnVR = −1.38, 95% CI: [−2.08, −0.78]) and ~3‐fold more at the inner bay disturbed versus the outer bay zone (lnVR = 0.96, 95% CI: [0.29, 1.63]) for *C. capistratus*, with no significant difference between inner and outer bay (lnVR = −0.43, 95% CI: [−1.11, 0.19]) (Figure 2b). For *H. puella*, lnVR values showed no significant differences, although there was a non‐significant trend towards higher variability at the inner bay disturbed zone (inner bay: lnVR = −0.23, 95% CI: [−0.85, 0.29]; outer bay: lnVR = 0.21, 95% CI: [−0.39, 0.93]) (Figure 2d).

### 3.3 Otolith‐based fish age determination and growth rate estimation

**FIGURE 3:**
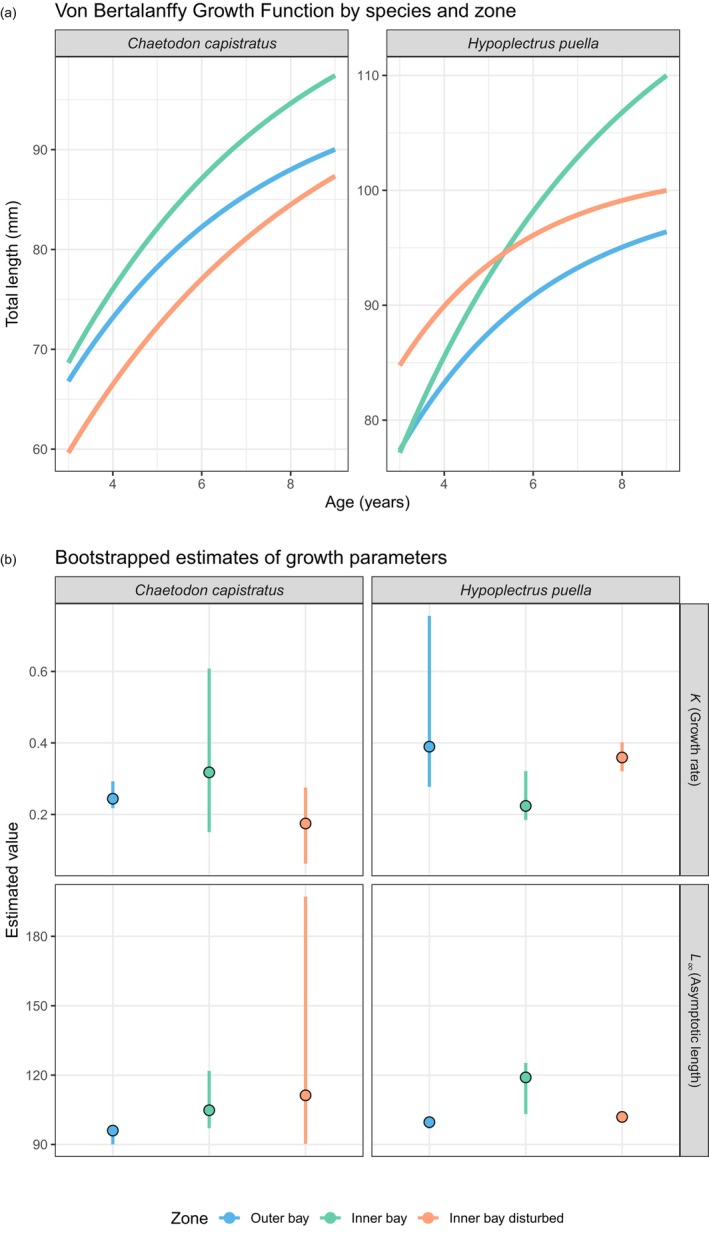
Comparison of fish growth between reef zones for the two fish species. (a) Growth curves among three zones for both study species from fitting the von Bertalanffy growth function (VBGF) and (b) estimates of bootstrapped growth parameters: Points depict bootstrapped mean parameter estimates and error bars indicate 95% confidence intervals. The age data were estimated from otolith analysis (*C. capistratus* *, N* = 158; *H. puella*,* N* = 127) and a total of five outliers were removed from the dataset for fitting growth curves resulting in 280 observations.

Fish age ranged from 3 to 9 years (mean ± SD = 5.8 ± 1.09) for *C. capistratus*, and from 3 to 8 years (5.1 ± 0.94) for *H. puella*. On average, *Chaetodon* individuals were oldest in the inner bay zone (6.41 ± 1.42) in comparison to both the disturbed (5.46 ± 0.81) and outer bay (5.66 ± 0.84) zones with a significant difference in age among zones (Kruskal; *χ* ^2^ = 13.56, *p* = 0.001; inner bay vs. inner bay disturbed zone: Dunn's adjusted *p* < 0.001; inner bay vs. outer bay zone: adjusted *p* = 0.01; inner bay disturbed vs. outer bay zone: adjusted *p* = 0.28). *Hypoplectrus* individuals in our sample exhibited a similar age structure across the three zones: inner bay (mean ± SD = 5.1 ± 0.82), inner bay disturbed (5.07 ± 1.01) and outer bay (5.14 ± 1) showing no significant difference in age among zones (Kruskal; *χ* ^2^ = 0.13, *p* = 0.94). Based on fish growth rate (*K*), *C. capistratus* grew fastest at the outer bay zone followed by the inner bay and slowest at the inner bay disturbed zone. In contrast, *H. puella* grew fastest in the inner bay disturbed and slowest in the inner bay zone (Figure 3a, Table 1). However, bootstrapped predictions of *K* did not significantly differ among zones for both species indicated by overlapping bootstrapped 95% confidence intervals (Figure 3b, Table S6). Estimates of asymptotic length (*L*_∞_) suggested that growth potential is greatest in the inner bay zone for both species and smallest at the outer bay (Figure 3a, Table 1). Predicted estimates of asymptotic length (*L*_∞_) were significantly different between all pairs of zones for *H. puella* (non‐overlapping bootstrapped 95% confidence intervals), but not *C. capistratus*, suggesting high variability in growth potential (*L*_∞_) despite more similar levels of growth rates (*K*) across zones (Figure 3b, Table S6).

**TABLE 1:** Fish growth by zone.

Species	Zone	*L_∞_*	*K*	*t*_0_
*Chaetodon capistratus*	Inner bay	110.47	0.19	−2.00
*Chaetodon capistratus*	Inner bay disturbed	102.27	0.17	−2.00
*Chaetodon capistratus*	Outer bay	97.86	0.23	−2.00
*Hypoplectrus puella*	Inner bay	125.23	0.19	−2.00
*Hypoplectrus puella*	Inner bay disturbed	102.04	0.36	−2.00
*Hypoplectrus puella*	Outer bay	100.28	0.30	−2.00

*Note*: Von Bertalanffy growth function (VBGF) parameters for *C. capistratus* (*N* = 158) and *H. puella* (*N* = 127) at each zone. *L*_∞_ is the asymptotic length or length infinitive, *K* is the growth rate at which *L*_∞_ is approached and *t*_0_ is the (hypothetical) point in time at which an individual is of length zero.

### 3.4 Diet composition

#### 3.4.1 Sequence analysis

**FIGURE 4:**
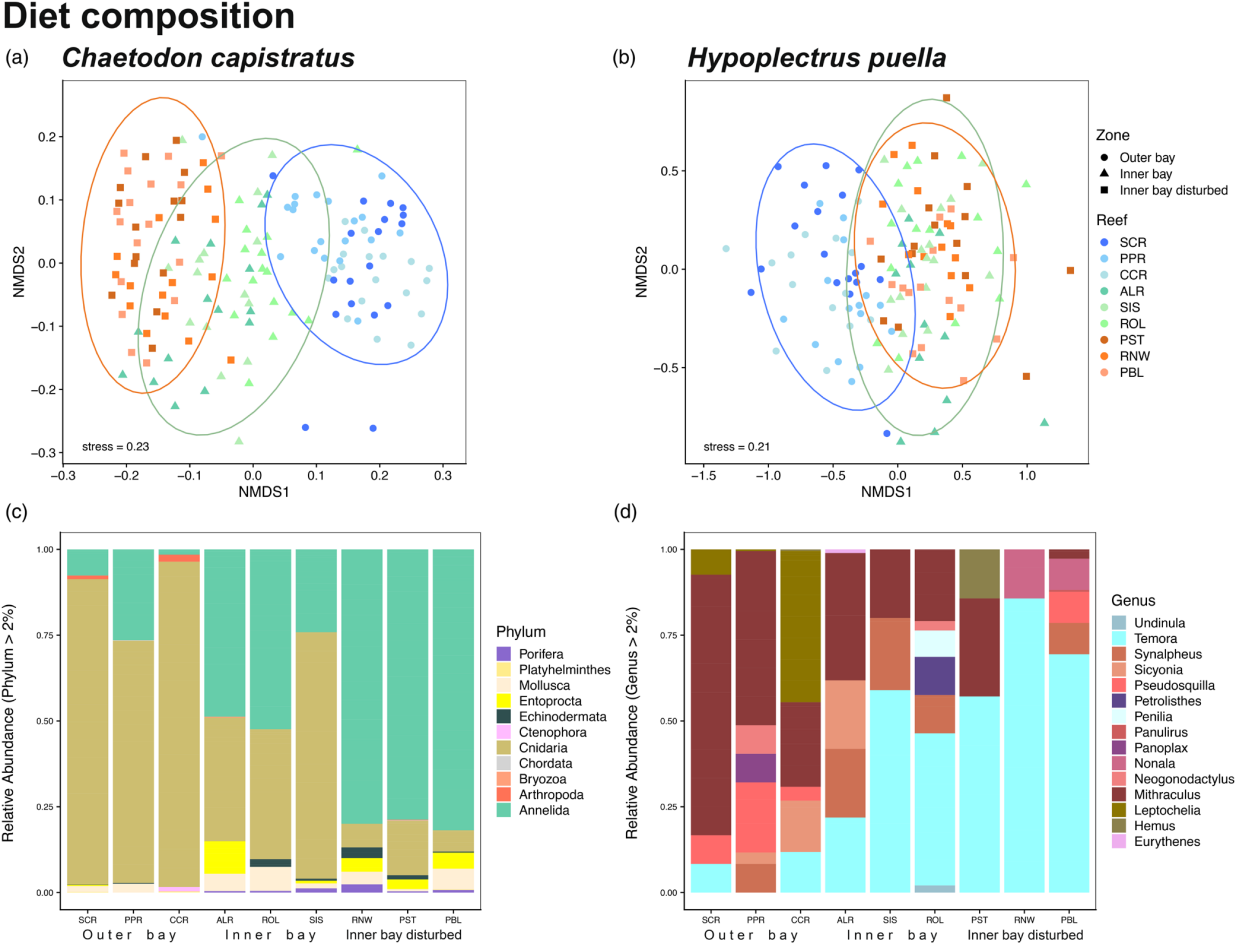
Differences in fish diet composition based on stomach and gut content metabarcoding across reefs. Nonmetric multidimensional scaling (NMDS) plots are based on Bray‐Curtis distance matrices between individuals of (a) *Chaetodon capistratus* and (b) *Hypoplectrus puella*. Dots depict fish individuals; reef zones are coded by shapes and colour: Blue = outer bay, green = inner bay, and orange = inner bay disturbed. Variation in diet composition across the habitat zones for (c) *C. capistratus* by phylum and (d) *H. puella* at the genus level within arthropods, their primary prey.

A total of 18,427,824 raw paired‐end reads were obtained. After denoising, removing chimeras, and processing, retained high‐quality reads clustered into 1009 OTUs assigned to the kingdom Metazoa, of which 166 (16.5%) were matched to species. An additional 613 OTUs (60.8%) could be assigned to higher taxonomic levels. The extraction and PCR controls did not show contamination. Steep sample‐based rarefaction curves indicate that the two target species consume a large diversity of prey in each reef zone that exceeds the prey we identified in our sampling (Figure S8A–C). The unimodal distribution of sequence read counts per sample (sequencing depth) peaked at approximately 25,000 reads for *C. capistratus* and at 6000 reads for *H. puella* (Figure S9A,B). Non‐metric multidimensional scaling (NMDS) of sequence read relative prey abundance data showed that *C. capistratus* from high coral cover reefs at the outer bay grouped together and was clearly separated from fish at the most degraded inner bay disturbed reefs (Figure 4a). Presence‐absence data showed a similar pattern; however, samples of the inner bay and inner bay disturbed zone were more similar in comparison to the relative abundance data (Figure S10A). The diet composition of *H. puella* at the outer bay zone separated from both the inner bay and inner bay disturbed zones for both relative abundance and presence–absence data, while there was no separation between the two zones located inside the bay (Figure 4b, Figure S10B). PERMANOVA confirmed significant differences in diet composition among reef zones for both species (Table S7) and pairwise testing confirmed that differences were significant between all pairs of zones for both study species, with the exception of *H. puella*'s diet composition between the inner bay and inner bay disturbed zone when considering relative abundance (Table S8). Diet composition of *C. capistratus* was dominated by cnidarians at the outer bay high coral cover sites, whereas its diet was dominated by annelids at the inner bay disturbed zone with a more mixed diet at the inner bay zone (Figure 4c, Figure S11A). Within the phylum Cnidaria, fish at the outer bay preferentially fed upon hard corals in the family Poritidae together with soft corals (Plexauridae, Gorgoniidae, Briareidae), while anemones and *Porites* sp. were dominant diet items at the inner bay zone (Figure S11B). In contrast, *Porites* sp. was less consumed at the inner bay disturbed zone, where sequence reads of both families Mussidae and Merulinidae and anemones (families: Aiptasiidae, Boloceroididae, Discosomatidae) were higher (Figure S11B). Soft corals were present in negligible proportions in the diets of fish residing at both zones inside the bay (Figure 4c, Figure S11A), while jellyfish appeared in the diet of fish in the inner bay disturbed zone (Figure S11B). Across all zones, there were low proportions of reads belonging to Corallimorpharia and Zoantharia (Figure S11A).

The diet of *H. puella* was dominated by arthropods at the phylum level across all reefs and zones (Figure S12A). However, differences in diet composition among reefs and zones emerged at lower taxonomic levels. Within Arthropoda, more copepods were consumed inside the bay and more decapods at the high coral cover outer bay reefs (Figure S12B). When considering prey communities at the genus level, macrocrustaceans dominated the diet at outer bay reefs and microcrustaceans, many of them planktonic taxa, were prevalent in the diet across the inner bay and inner bay disturbed zones (Figure 4d). At both zones located inside the bay, *H. puella*'s arthropod diet contained a large proportion of the copepod *Temora stylifera*, whereas diets at outer bay reefs were dominated by crabs in the genus *Mithraculus*, and to a lesser extent tanaid crustaceans (genus: *Leptochelia*) and mantis shrimp (genus: *Pseudosquilla*) (Figure 4d), whereas prawns (genus: *Sicyonia*), rubble crabs (genus: *Panoplax*), snapping shrimp (genus: *Synalpheus*) and mantis shrimp (genus: *Neogonodactylus*) were consumed in smaller proportions (Figure 4d). At the phylum level, Chordata constituted the second most important diet item of *H. puella* but relative read abundances were significantly lower than for Arthropoda (Figure S12A). Chordates largely consisted of fishes (Figure S13A), with Gobiiformes being most frequently consumed followed by Blenniiformes (Figure S13A). COI metabarcoding detected a broad taxonomic range of fishes (51 OTUs, class: Actinopterygii), of which 19 were identified at the species level (Table 2). The chaenopsid blenny *Emblemariopsis arawa*k was the most frequently detected species (at six of nine reefs) (Figure S13B).

**TABLE 2:** Fish prey species.

Class	Order	Family	Genus	Species	Common name
Actinopterygii	Acanthuriformes	Acanthuridae	*Acanthurus*	*Acanthurus chirurgus*	Doctor fish
Actinopterygii	Kurtiformes	Apogonidae	*Phaeoptyx*	*Phaeoptyx xenus*	Sponge cardinalfish
Actinopterygii	Kurtiformes	Apogonidae	*Phaeoptyx*	*Phaeoptyx pigmentaria*	Dusky cardinalfish
Actinopterygii	Blenniiformes	Blenniidae	*Hypleurochilus*	*Hypleurochilus geminatus*	Crested blenny
Actinopterygii	NA	Centropomidae	NA	NA	Snook
Actinopterygii	Blenniiformes	Chaenopsidae	*Acanthemblemaria*	*Acanthemblemaria chaplini*	Papillose blenny
Actinopterygii	Blenniiformes	Chaenopsidae	*Emblemariopsis*	*Emblemariopsis arawak*	Araw glass blenny
Actinopterygii	Gobiiformes	Gobiidae	*Coryphopterus*	*Coryphopterus glaucofraenum*	Bridled goby
Actinopterygii	Gobiiformes	Gobiidae	*Elacatinus*	*Elacatinus illecebrosus*	Barsnout goby
Actinopterygii	Gobiiformes	Gobiidae	*Coryphopterus*	*Coryphopterus personatus*	Masked goby
Actinopterygii	Gobiiformes	Gobiidae	*Coryphopterus*	*Coryphopterus eidolon*	Pallid goby
Actinopterygii	Gobiiformes	Gobiidae	*Risor*	*Risor ruber*	Tusked goby
Actinopterygii	Gobiiformes	Gobiidae	*Gnatholepis*	*Gnatholepis thompsoni*	Goldspot goby
Actinopterygii	Lutjaniformes	Haemulidae	*Haemulon*	*Haemulon macrostomum*	Spanish grunt
Actinopterygii	Lutjaniformes	Haemulidae	*Haemulon*	*Haemulon steindachneri*	Latin grunt
Actinopterygii	Gymnotiformes	Hypopomidae	*Brachyhypopomus*	*Brachyhypopomus occidentalis*	Bluntnose knifefish
Actinopterygii	Labriformes	Labridae	*Sparisoma*	*Sparisoma chrysopterum*	Redtail parrotfish
Actinopterygii	Blenniiformes	Labrisomidae	*Starksia*	*Starksia occidentalis*	Occidental blenny
Actinopterygii	NA	Sciaenidae	NA	NA	Drum
Actinopterygii	Perciformes	Serranidae	*Serranus*	*Serranus flaviventris*	Twinspot bass
Actinopterygii	Blenniiformes	Tripterygiidae	*Enneanectes*	*Enneanectes altivelis*	Lofty triplefin

*Note*: Fishes identified in the diet of *H. puella* (including only those OTUs that were identified to at least family level, 41% of all 51 OTUs assigned to Actinopterygii).

### 3.5 Relationship between coral cover and fish diet

**FIGURE 5:**
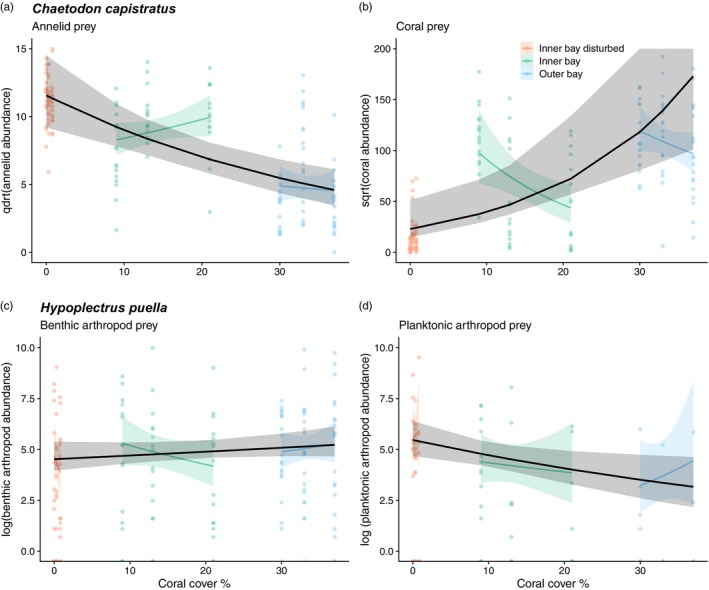
Effects of coral cover on fish diets. Negative binomial generalized linear mixed models (GLMMs) were fitted to predict the effect of percent coral cover on the sequencing read abundance of dominant diet items consumed by two fish species: *Chaetodon capistratus* feeding on (a) annelids and (b) hard coral, and *Hypoplectrus puella* feeding on (c) benthic arthropods and (d) planktonic arthropods. Black lines depict model predictions across levels of percent coral cover with 95% confidence intervals (areas shaded in grey) based on the fixed effects from the GLMMs. Smoothed coloured lines with 95% confidence intervals (CIs) (coloured shaded areas) represent variability within each reef zone based on raw data. The contrasting patterns between black and coloured lines suggest the presence of scale‐dependent trends in prey consumption. Annelid read abundance was fourth root transformed, hard coral read abundance was square root transformed and reads of both arthropod groups were log transformed to improve model fitting.

Coral cover was a strong predictor of the relative abundance of hard corals and annelids in the diet of *C. capistratus* (Figure 5a,b, Table 3). Reef zones varied in percent coral cover, and there was a significant relationship between the butterflyfish diet and zones (Table 3), with annelids decreasing and corals increasing in the diet when moving from inner bay disturbed zone to the inner bay zone and the outer bay zone. Coral cover did not predict the abundance of the dominant prey of *H. puella*, benthic arthropods (Figure 5c, Table 3), but it did predict consumption of planktonic arthropods (Figure 5d, Table 3). We found no significant relationship between coral cover and the abundance of fishes detected within the guts of *H. puella* (Table 3). In some cases, within‐zone trends in read abundances were opposite to the overall model slopes (Figure 5a,b,d). In addition, for both fish species we detected no significant effect of fish age on the proportions of main prey items in the diets (Table 3).

**TABLE 3:** Effects of coral cover on fish diets.

Species	Model	Response (Diet)	Predictor	Random Effect	*χ* ^2^	*p*
*Chaetodon capistratus*	1	Annelid	Coral cover	Zone/Reef	5.94	**0.015**
	2	Annelid	Age	Zone/Reef	0.233	0.629
	3	Hard coral	Coral cover	Zone	5.38	**0.02**
	4	Hard coral	Age	Zone/Reef	0.034	0.854
*Hypoplectrus puella*	5	Benthic arthropods	Coral cover	Zone/Reef	1.73	0.188
	6	Benthic arthropods	Age	Zone/Reef	0.859	0.354
	7	Planktonic arthropods	Coral cover	Zone/Reef	4.62	**0.032**
	8	Planktonic arthropods	Age	Zone/Reef	0.04	0.843
	9	Fish	Coral cover	Zone/Reef	0.77	0.38
	10	Fish	Age	Zone/Reef	0.18	0.66

*Note*: Results from separate generalized linear mixed models (GLMMs) for each dominant prey item, testing the effects of coral cover and fish age on prey sequencing read abundance in the diets of *Chaetodon capistratus* and *Hypoplectrus puella*. For each diet item, independent models were run to test the effects of coral cover and age as predictors. The random effect structure was determined by the study design: Zone/reef for non‐coral items, and zone only for hard coral to avoid collinearity with coral cover. Significant effects are depicted in bold.

### 3.6 Diet strategy

**FIGURE 6:**
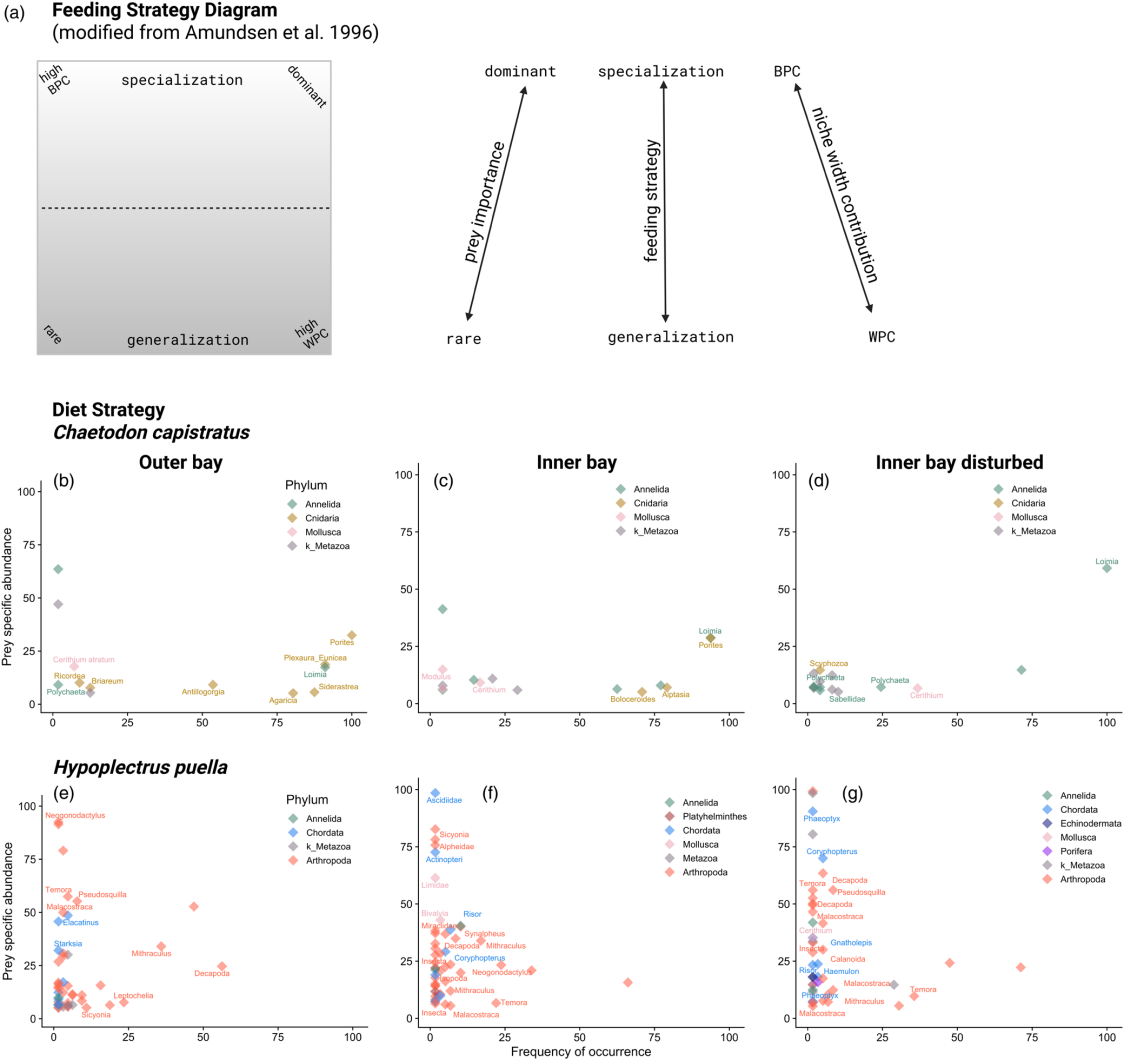
Fish feeding strategies across the habitat zones with different levels of coral cover. (a) Schematic diagram modified from Amundsen et al. (1996) illustrating how feeding strategy as shaped by niche width contribution and prey importance is inferred from i the vertical axes indicating specialization (upper portion of plot) and generalization (lower portion of plot) and ii) the diagonal axis representing the within phenotype component (WPC, lower right corner) and between phenotype component (BPC, upper left corner) indices. (b–g) Graphical analysis of fish diet strategy at three reef zones using relative read abundance data for *C. capistratus* (b–d) and *H. puella* (e–g) following the method of Amundsen et al. (1996) based on and modified from Costello (1990). Points represent OTUs assigned to prey taxa. Points located in the upper right indicate a specialized diet at the population level (abundant and frequent diet items), whereas points in the lower left corner indicate opportunistic, occasional diet items that are found rarely and in low abundance in the diet. Frequency of occurrence = percentage of fish in which a prey item was present versus the total number of fish. Prey‐specific abundance = percentage of diet made‐up by a given prey item (OTU) across only the number of fish individuals where it occurred.

Amundsen plots of fish diet strategy (Figure 6a) across zones suggested that the diet of *C. capistratus* was dominated by very few prey items, as indicated by points located in the middle to upper right corner of the plots (Figure 6b–d). This relatively specialized diet was complemented by a diverse array of occasional prey items that were consumed in low abundance (lower left corner of the plot). While *C. capistratus* appeared as a facultative specialist, *H. puella* displayed a generalist diet that was dominated by arthropods across all zones (Figure 6e–g). Across the habitat zones, *C. capistratus* switched its main diet item from hard coral, that is, *Porites* sp. (phylum Cnidaria) at the outer bay zone (Figure 6b), to a mix of *Porites* sp. and a sessile worm, *Loimia medusa* (phylum Annelida) at the inner bay zone (Figure 6c), towards a diet dominated by *Loimia medusa* at the inner bay disturbed zone (Figure 6d). The observed switch in the main diet item entailed that the diet of individual fish was less diverse (i.e. more specialized) as indicated by a decrease in the within phenotype component (WPC) at the disturbed zone in comparison with the other two zones (Figure 6d). In the outer bay, *H. puella* consumed crabs in the genus *Mithraculus* frequently and in large quantities (25–50%) relative to other prey items, which were less apparent inside of the bay (<25%) (Figure 6e–g). In contrast, the frequency of microcrustaceans in the diet was higher in inner bay zones, dominated by copepods in the genus *Temora* (Figure 6f,g). At both inner bay zones, we found that the diet among individuals was more variable as indicated by an increase in the between phenotype component (BPC), implying an increase in individual specialization and a broader diet on the population level at these reefs (Figure 6f,g) relative to outer bay reefs (Figure 6e).

## 4 DISCUSSION

Our study was conducted across three habitat zones featuring different levels of coral cover and diversity that represented degrees of reef degradation associated with variation in invertebrate prey assemblages. While the disturbance history is undoubtedly responsible for differences in benthic community structure, other factors such as wave exposure, water circulation, and human population activities likely play an important role as well. These factors may covary with coral cover and potentially contribute to differences in prey availability. Against this backdrop we showed that the diet composition of two benthic‐feeding coral reef fish species (*C. capistratus* and *H. puella*) with distinct feeding strategies was influenced by the proportion of live coral cover. Combining high‐resolution diet data with ecological survey data and otolith analysis revealed relationships between resource use, benthic cover, and fish condition and growth. We found dietary differences in both fish species between healthy and degraded reefs; however, the extent to which dietary adjustments shield potentially adverse effects of spatial differences in prey availability varied between fish species, as evidenced by more variable body condition observed only in the browsing species inhabiting the most degraded reefs. Our results further suggest that fish trophic roles can vary within species across small spatial scales in relation to coral cover.

### 4.1 Changes in fish diet with reef degradation

Contrary to our expectation, *C. capistratus* did not broaden its diet where coral cover was low, but switched preference while narrowing its diet. It maintained its browsing feeding mode (i.e. moving between resource patches nipping on sessile prey) on degraded reefs by primarily feeding on terebellid worms (phylum: Annelida, class: Polychaeta) resembling the coral polyps that it typically consumes in providing an evenly distributed and abundant prey resource. This feeding behaviour potentially allows individuals to maintain a similar energy budget between healthy and degraded reefs (Uchida et al., 2007; Van Leeuwen et al., 2013). While *C. capistratus* has been previously reported to include polychaete tentacles in its diet (e.g. families: Serpulidae, Terebellidae), the percent volume detected in stomachs was commonly low in relation to cnidarian prey of largely hexacorals and octocorals. For example, Birkeland and Neudecker (1981) showed that *C. capistratus* can complement its anthozoan dominated diet (80%) with items of high nutritional value such as polychaete worms (Rotjan & Lewis, 2008), but no previous study has shown a near complete switch. Prey may need to exceed a certain abundance threshold to represent a diet item worth exploiting for *C. capistratus*, and previous studies from the 1980s were conducted on much less disturbed reefs. Our findings are consistent with previous studies finding *C. capistratus* to feed selectively (Birkeland & Neudecker, 1981; Casement, 2021; Gore, 1984; Lasker, 1985; Liedke et al., 2018). *Chaetodon capistratus* specialized in chemically defended worm tentacles at degraded reefs, indicating an adaptation to feeding on chemically defended prey. This dietary specialization may serve to avoid competition with the Caribbean congeners *C. striatus* and *C. ocellatus*, which are known to be less tolerant of dietary allelochemicals (Liedke et al., 2018; Pitts, 1991; Vrolijk et al., 1995), thus highlighting a potential ecological advantage in resource utilization within degraded reef environments.

*Hypolplectrus puella* likely fed in relation to resource availability as both the consumption and relative density of decapod crustaceans decreased at the inner bay disturbed zone. Despite their small size, copepods potentially provide a more numerous and evenly distributed food source than crabs under degraded conditions. However, as calanoid copepod distribution has been shown to be uniform across our study area (Rodas et al., 2020), the observed dietary pattern was likely not driven by relative plankton availability, but rather changes in the accessibility of macrocrustacean prey. In contrast to the different crustacean prey (macro vs. microcrustaceans), the contribution of fishes in the diet of *H. puella*, which constituted the second most common diet item, did not vary with coral cover. Overall, the relative proportion of fish prey consumed by *H. puella* resembled that of its congener *H. unicolor* (the butter hamlet) at our study area, and was thus higher than previously reported (Puebla et al., 2018). Our metabarcoding approach likely surpassed previous visual analyses that might have underestimated the proportion and diversity of fish prey in the diet of *H. puella* (~10%; Puebla et al., 2018; Randall, 1967; Whiteman et al., 2007) as fish can be digested faster than crustaceans (e.g. four times faster in rock cod, Beukers‐Stewart & Jones, 2004). In addition, *H. puella* targeted fish species that were previously not detected in *H. unicolor*'s diet at Bocas del Toro and seemed to consume proportionally more crabs than its congener. Previous diet analyses, in contrast, reported high levels of dietary overlap among hamlet species suggestive of ecological equivalence (Holt et al., 2008; Whiteman et al., 2007). However, confirming whether these two species might partition their prey to some extent requires further study with comparable methodologies.

Interestingly, for both species the level of consumption of particular prey followed contrasting trends across versus within zones in some cases. For example, the sequencing read abundance of annelids in *C. capistratus'* diet decreased with declining coral cover in the inner bay zone while it generally increased across zones. This may be due to much larger differences in coral cover between zones than within zones and to local habitat effects on diet not captured by overall models.

### 4.2 Changes in fish condition with reef degradation

Our results suggest that low coral cover reefs potentially provide less suitable resources for *C. capistratus*, whereas *H. puella* clearly appears more resilient. While there was no significant difference in body condition among zones, we found significantly more variable condition estimates (*K*_n_) between *C. capistratus* individuals on reefs of the disturbed inner bay. This effect was still strong after controlling for fish size (TL) suggesting that other factors such as resource limitation may lead to divergent responses in fish individuals. *C. capistratus* individuals have previously been found to exhibit more variable gut microbiome composition on degraded reefs of the Bay of Almirante compared to fish in zones with higher coral cover suggesting more diverse diets and possible physiological stress leading to dysbiosis (Clever et al., 2022). Interestingly, condition significantly declined with increasing fish size in the inner bay zone where fish also reached their largest sizes and were oldest in comparison to both other zones. This may reflect difficulty in meeting energetic demands beyond a certain size, for example, related to increased metabolic costs, reproductive investment, competition or limited resources. Another explanation might be that fish in the inner bay zone grew older due to the combination of sufficient live coral cover compared to the inner bay disturbed zone, and potentially lower predation pressure than at the outer bay zone where coral cover was highest and fishing pressure likely lower, potentially implying higher predation pressure on butterflyfishes (Cramer, 2013; Seemann et al., 2018). Conspicuously, the density of *C. capistratus* did not significantly vary among zones. The population size of versatile feeders may only slowly respond to changes in prey availability; for example, the most specialized species of Indo‐Pacific butterflyfishes, but not the more generalist species, were shown to have population sizes limited by resource availability (Lawton & Pratchett, 2012). Some of the few previous studies examining the effects of coral reef habitat on fish body condition found reduced levels associated with dietary changes (Berumen et al., 2005; Hempson et al., 2017; Pratchett et al., 2004). Although *C. capistratus* densities were stable across zones, long‐term sublethal effects of habitat degradation may manifest over time.

Our data suggest that the condition of *H. puella* did not significantly decline with decreasing coral cover despite the marked difference in size between its crustacean prey across the habitat zones, demonstrating a versatile nutritional and behavioural physiology. However, while condition increased with fish size at the outer bay, it decreased at the inner bay disturbed zone where fish were significantly larger and heavier. These findings suggest that energetic constraints (e.g. from reliance on small prey) may compromise the condition of *H. puella* in larger individuals at degraded reefs. In contrast, a macrocrustacean diet may better support larger individuals at the outer bay, while other factors such as predation pressure or exposure that may increase energy demands at this zone may limit density and growth. The size ratio between fish and its prey influences the effort needed for searching prey and the relative contribution of a prey to a predator's energy needs (Hart & Gill, 1993). Feeding on planktonic prey suggests low search effort but also low caloric return per prey especially in large individuals (Hart & Gill, 1993). Apart from possible energetic constraints for large individuals, the increased reliance of *H. puella* on an alternative food source potentially providing lower nutritional value (Hart & Gill, 1993) did not translate into lower mean density, body condition or growth rate at the inner bay disturbed zone, suggesting the ease of obtaining plankton made up for its relatively low source of energy. Interestingly, mean density and asymptotic length were greatest at the inner bay zone indicating favourable conditions for long‐term growth and a preference for sheltered inner bay habitats as shown previously (Hench et al., 2017). Overall, in contrast to previous results showing that shifted diets on degraded reefs led to less energy stored in the livers of another coral reef mesopredator (Hempson et al., 2018), our results suggest that *H. puella* is able to cope with degraded reef conditions. Here we considered levels of condition and growth using relatively coarse resolution metrics, that is, an index of fish condition (Le Cren's relative condition factor, *K*_n_) and age determination from otoliths based on numbers of rings (not considering ring sizes or distance from a ring to the otolith margin). Length–weight based condition indices such as *K*_n_—while widely used in fisheries—remain proxies and thus less accurate than approaches directly assessing organismal parameters such as fat storage in liver tissue (e.g. Hempson et al., 2017; Pratchett et al., 2004), or RNA:DNA ratios (Liedke et al., 2016). We acknowledge that processes beyond the scope of this study (e.g. activity levels, reproduction) likely moderate levels of condition and growth.

### 4.3 Implications for management and conservation

While most pronounced in *C. capistratus*, both species differed in their trophic functions across habitat zones thanks to flexible feeding behaviours, which may shape food webs in different ways at healthy versus degraded reefs. The predominant prey items of *C. capistratus* and *H. puella* at high coral cover reefs (live corals and macrocrustaceans, respectively) rely upon planktonic carbon sources and symbiotic photosynthesis in the case of corals, and epibenthic food in the case of many crustaceans. At degraded reefs in contrast, terebellid worms and calanoid copepods use detrital deposits and phytoplankton, respectively. This implies that both fish species used different trophic pathways at healthy and degraded reefs. Habitat degradation in the form of fragmentation and land use change has previously been shown to lead to a simplified food web structure in tidal creeks in the Bahamas and a freshwater system in Croatia (Layman et al., 2007; Price et al., 2019). The reliance of *H. puella* on planktonic food in the disturbed zone was in line with previous findings suggesting that pelagic food sources may increasingly support coral reef fishes on degraded reefs (Morais & Bellwood, 2019). Further investigation of prey diets or verification with other trophic markers (e.g. compound‐specific stable isotopes; McMahon et al., 2016) could further substantiate carbon sources.

Our study adds to recent work on how consumer‐resource interactions alter coral reef food webs in response to degradation at relatively small spatial scales (Hempson et al., 2018; Karkarey et al., 2017; Layman et al., 2007; Semmler et al., 2022), thereby influencing energy flow and ultimately ecosystem functioning (Duffy et al., 2007). Species interactions are thought to underpin stabilizing mechanisms, such as functional redundancy (Rosenfeld, 2002) and trophic compensation (e.g. Ghedini et al., 2015), under conditions of environmental change. Yet this notion is being increasingly scrutinized in the case of coral reefs, where high levels of niche partitioning may render coral reef fish assemblages more vulnerable than previously assumed (Bejarano et al., 2017; Brandl et al., 2020; Brandl & Bellwood, 2014; Kramer et al., 2015; Leray et al., 2015; Semmler et al., 2021). Intraspecific dietary variation as documented by the present study may play a role in mediating this pattern (Albert et al., 2010) and thus potentially affect levels of functional redundancy within the fish assemblage at a given location. In addition, our findings question the usefulness of coarse trophic classifications of species that largely lack empirical ground‐truthing. For example, Parravicini et al. (2020) found coral reef fish invertivores were more diversified than suggested by previous classifications. Our study corroborates their findings and improves the resolution of benthic invertivore diets on coral reefs.

Our finding that *C. capistratus* exhibited increased variability in condition at degraded reefs and greater dietary variation among reef zones in contrast to *H. puella*, suggests that benthic invertivorous fishes that specialize on sessile taxa may be more susceptible to reef degradation than those that specialize on free‐living invertebrates. However, energetic trade‐offs on degraded reefs may constrain condition for both feeding strategies, particularly for larger individuals. High‐resolution DNA‐based analysis revealed that within‐species dietary variation was greater than previously thought for our study species. We further demonstrated that versatile feeding behaviour could entail the use of different trophic pathways between high and low coral cover reefs. Consequently, we advocate taking into account diet versatility and limitations on body condition in putatively generalist strategies, as they can influence both species persistence and tropho‐dynamics on degrading coral reefs.

## AUTHOR CONTRIBUTIONS

Friederike Clever, Matthieu Leray and Richard F. Preziosi conceived and designed the study. Friederike Clever and Matthieu Leray conducted the fieldwork. Friederike Clever dissected the fish stomachs and guts and extracted the DNA. Friederike Clever and Matthieu Leray prepared the DNA for sequencing. Bryan Nguyen processed the sequencing data. Brígida De Gracia, Aaron O'Dea, and Helio Quintero Arrieta conducted the otolith analysis. Richard F. Preziosi, Nancy Knowlton, Andrew H. Altieri, W. Owen McMillan, Aaron O'Dea, and Matthieu Leray contributed reagents and supplies. Friederike Clever analysed the data and wrote the first draft of the manuscript with input from Matthieu Leray, Richard F. Preziosi, and Andrew H. Altieri. Brígida De Gracia, Aaron O'Dea, Nancy Knowlton, and W. Owen McMillan contributed to a later version of the manuscript. All authors reviewed the manuscript, contributed to the final version and provided final approval for publication.

## CONFLICT OF INTEREST STATEMENT

The authors declare no conflict of interest.

## Supporting information


**Figure S1.** Determination of otolith incremental rings.
**Figure S2.** Coral diversity across reefs and zones.
**Figure S3.** Benthic composition.
**Figure S4.** Differences in fish community structure.
**Figure S5.** Mean densities of invertebrate prey taxa.
**Figure S6.** Benthic invertebrate community composition.
**Figure S7.** Fish body condition among zones.
**Figure S8.** Sample‐based rarefaction curves.
**Figure S9.** Sequencing depth by samples and species.
**Figure S10.** Differences in fish diet composition based on dietary metabarcoding among zones.
**Figure S11.** Diet composition of *Chaetodon capistratus*.
**Figure S12.** Diet composition of *Hypoplectrus puella*.
**Figure S13.** Diet composition of *Hypoplectrus puella* (fish prey).
**Table S1.** The versatile COI primer pair (Geller et al., 2013; Leray et al., 2013) that was used in this study.
**Table S2.** Species‐specific blocking primer sequences for two coral reef fishes, *Hypoplectrus puella* and *Chaetodon capistratus*.
**Table S3.** Differences in invertebrate densities.
**Table S4.** Pairwise comparisons of invertebrate mean densities.
**Table S5.** Fish length and weight between zones.
**Table S6.** Comparison of growth predictions.
**Table S7.** Fish diet composition among zones.
**Table S8.** Pairwise comparisons of fish diet composition.

## Data Availability

Sequencing data have been submitted to the NCBI Short Read Archive (SRA) database (https://www.ncbi.nlm.nih.gov/sra) under BioProject Accession: PRJNA1365833. Raw data and R code are available from the Dryad Digital Repository https://doi.org/10.5061/dryad.qz612jmpz (Clever et al., 2025).
