# Supplementary material for: Dietary resilience of coral reef fishes to habitat degradation
Source: J Anim Ecol. 2025 Dec 14;95(3):397–417. doi: 10.1111/1365-2656.70196 (PMC12957732; doi:10.1111/1365-2656.70196)
Supplement: Supplementary file 1 — Figure S1. Determination of otolith incremental rings. Figure S2. Coral diversity across reefs and zones. Figure S3. Benthic composition. Figure S4. Differences in fish community structure. Figure S5. Mean densities of invertebrate prey taxa. Figure S6. Benthic invertebrate community composition. Figure S7. Fish body condition among zones. Figure S8. Sample‐based rarefaction curves. Figure S9. Sequencing depth by samples and species. Figure S10. Differences in fish diet composition based on dietary metabarcoding among zones. Figure S11. Diet composition of Chaetodon capistratus. Figure S12. Diet composition of Hypoplectrus puella. Figure S13. Diet composition of Hypoplectrus puella (fish prey). Table S1. The versatile COI primer pair (Geller et al., 2013; Leray et al., 2013) that was used in this study. Table S2. Species‐specific blocking primer sequences for two coral reef fishes, Hypoplectrus puella and Chaetodon capistratus. Table S3. Differences in invertebrate densities. Table S4. Pairwise comparisons of invertebrate mean densities. Table S5. Fish length and weight between zones. Table S6. Comparison of growth predictions. Table S7. Fish diet composition among zones. Table S8. Pairwise comparisons of fish diet composition. [file JANE-95-397-s001.docx]

Supplementary Information

**Dietary resilience of coral reef fishes to habitat degradation**

Friederike Clever, Richard F. Preziosi, Bryan Nguyen, Brígida De Gracia, Helio Quintero Arrieta, W. Owen McMillan, Andrew H. Altieri, Aaron O’Dea, Nancy Knowlton, and Matthieu Leray

Contents

Supplementary Figures 2

Supplementary Tables 15

Supplementary Methods 19

References 20

**Supplementary figures**


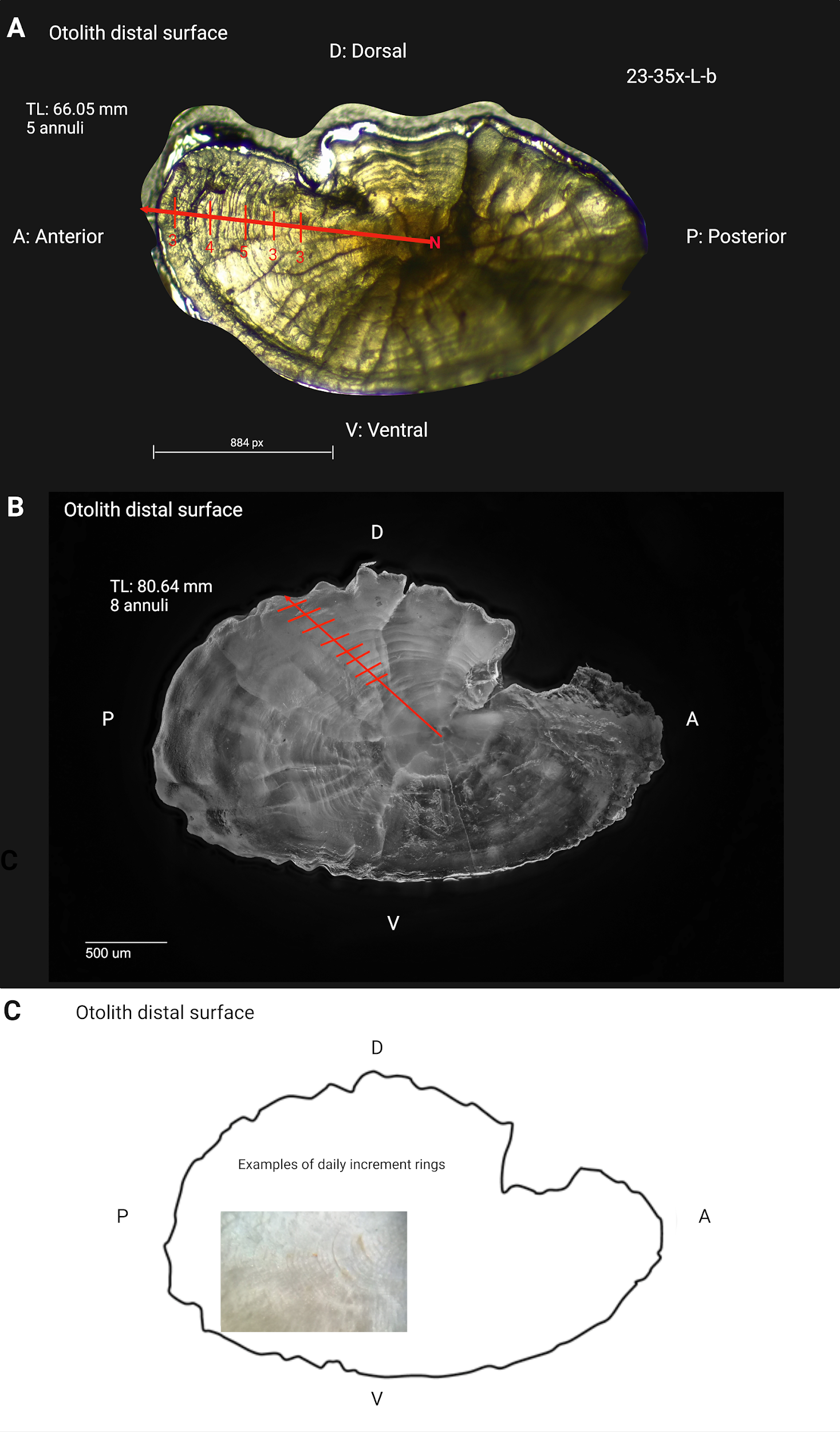


**Figure S1.** **Determination of otolith incremental rings.** Depicted are the distal surfaces of the (A) left and (B) right sagittal otolith of a *Chaetodon capistratus* fish individual; (C) right sagittal otolith with visible daily increments.

**
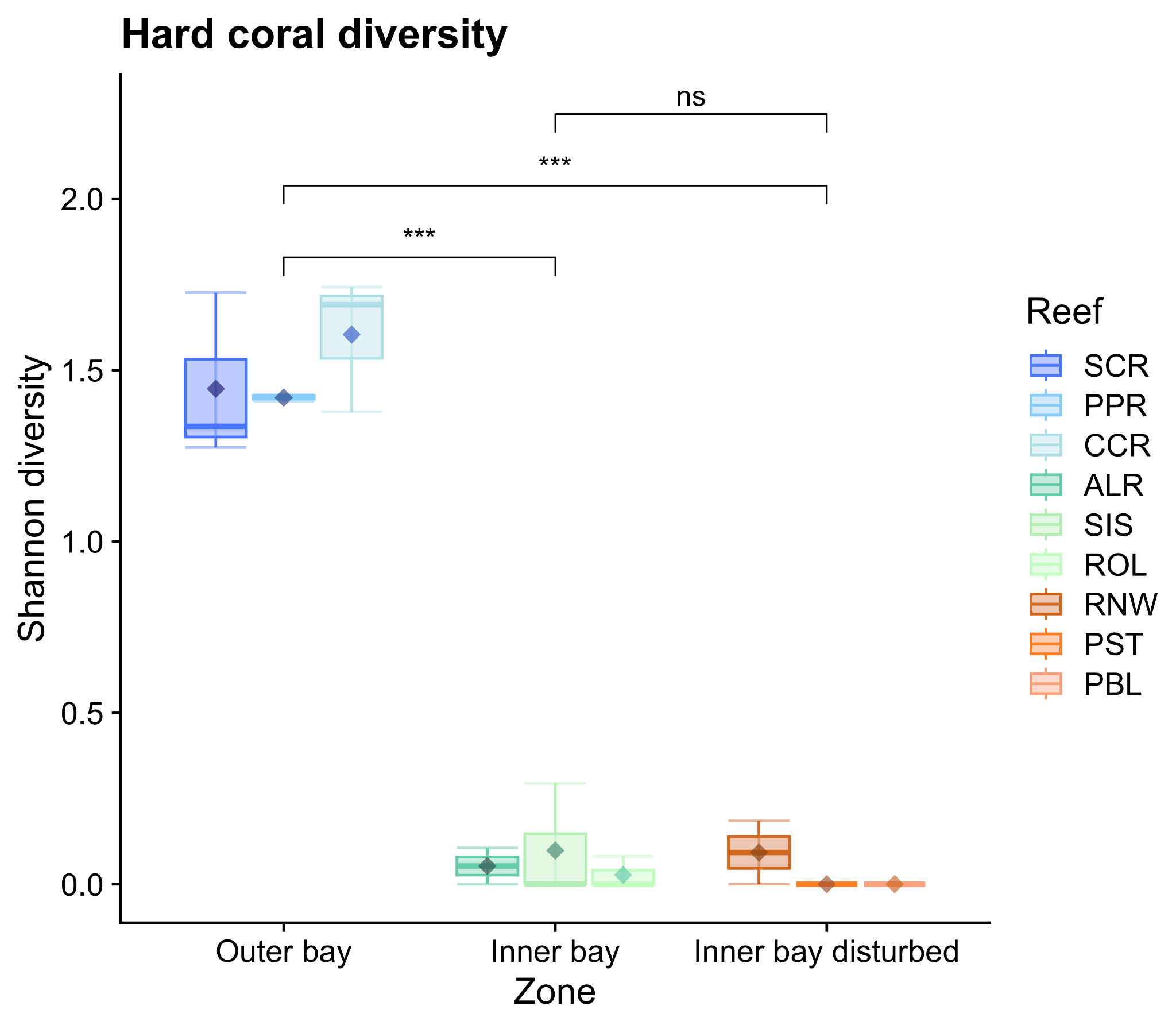
**

**Figure S2.** **Coral diversity across reefs and zones**. Estimates of hard coral diversity (including Scleractinian corals and *Millepora* spp.) at nine non-contiguous reefs and across three different reef zones at the Bahía Almirante, Bocas del Toro, Panamá. Diamonds depict means.

**
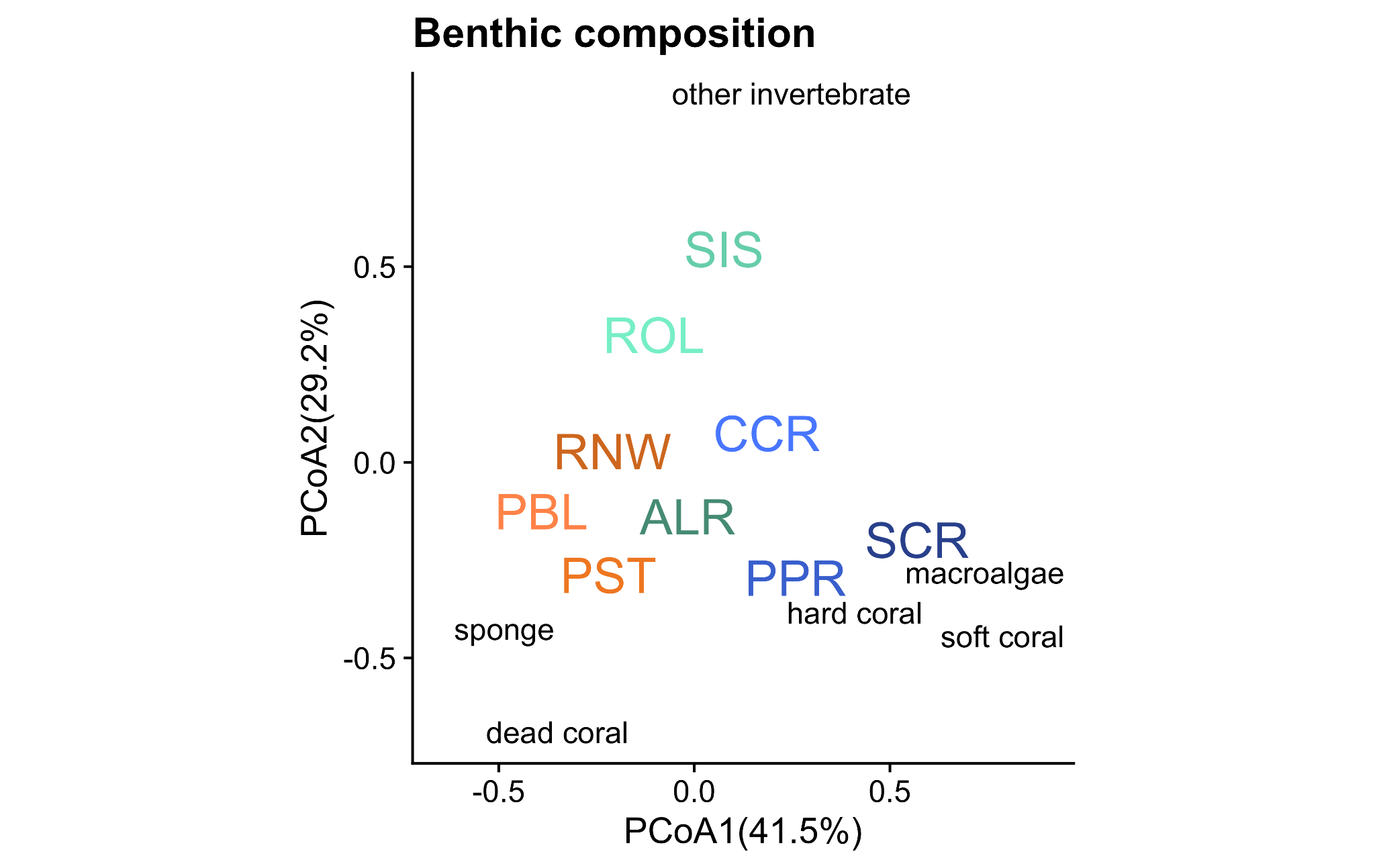
**

**Figure S3.** **Benthic composition.** Nine study reefs across three reef zones (blue = outer bay, green = inner bay, orange = inner bay disturbed) at the Bahìa Almirante, Bocas del Toro, Panamá, clustering according to differences in benthic composition. Also depicted are the major benthic groups or substrate types responsible for driving differences between the nine study reefs.


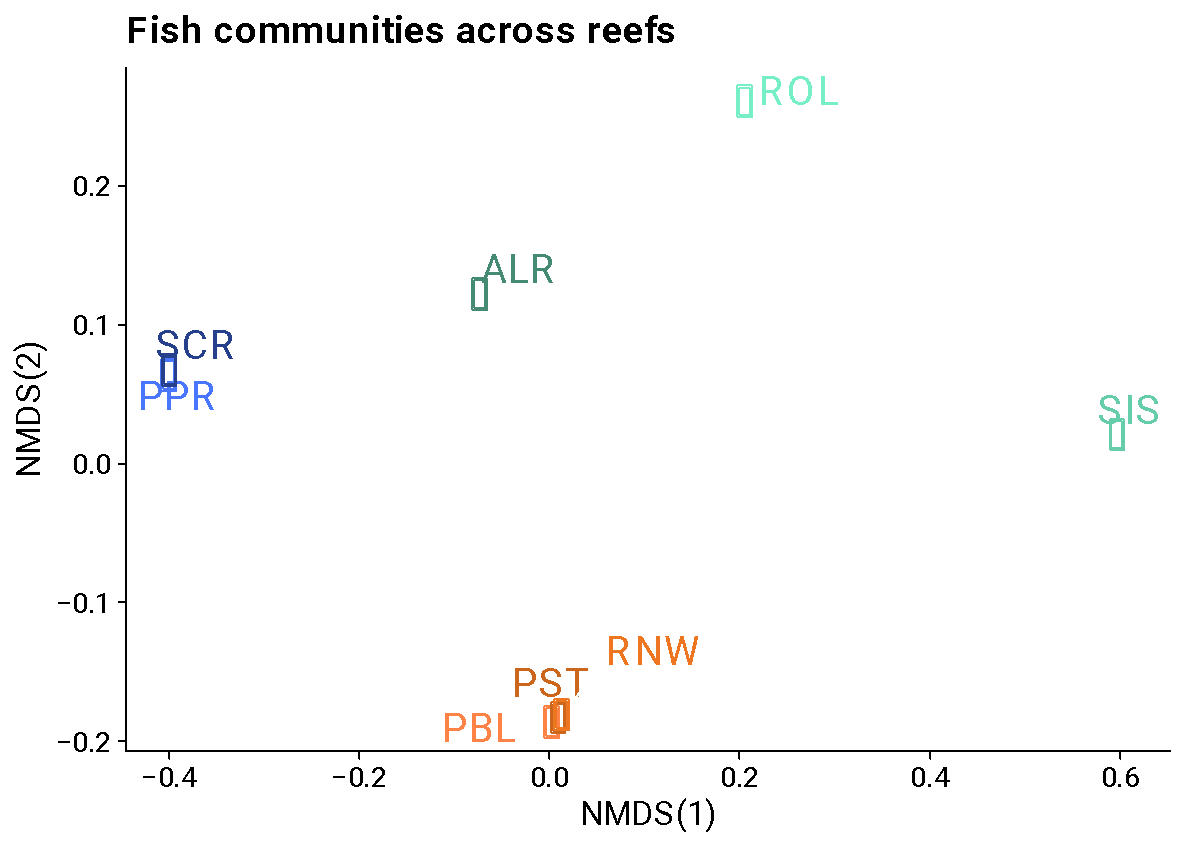


**Figure S4.** **Differences in fish community structure.** Nonmetric multidimensional scaling (NMDS) using Bray Curtis dissimilarity of fish communities across reefs and zones (colour coded): blue = outer bay, green = inner bay, red = inner bay disturbed. Fish surveys were conducted at all of our nine study reefs except for Coral Caye Reef (CCR) in the outer bay zone. The visual fish surveys included diurnal and non-cryptic species.

**Figure S5.** **Mean densities of invertebrate prey taxa.** Prey taxa of two focal fish species at our study area across three reef zones from highest coral cover (outer bay) to lowest coral cover (inner bay disturbed). Depicted are mean densities of (A) all recorded benthic macro-invertebrates (> 2 mm); (B) terebellid worms, a main diet item of *C. capistratus*; (C) Arthropods, the main prey of *H. puella*. In addition, we examined lower taxonomic levels within the phylum Arthropoda to examine potential differences among zones that might influence fish diet: (D) decapod crustaceans; (E) brachyuran crabs and (F) mithracid crabs. Macro-invertebrates (> 2 mm) were collected within three quadrats per reef (50 x 50 cm) and on three reefs per zone.

**Figure S6.** **Benthic invertebrate community composition.** (A) Invertebrate relative densities collected using quadrats on dead patches (predominantly *Agaricia tenuifolia*) at eight study reefs depicted at the taxonomic level of class; where the level of class was not identified, the next higher taxonomic level identified is depicted indicated by p = phylum or sub_p = subphylum. (B) the relative densities of arthropod taxa collected from the same quadrats as (A).

**Figure S7.** **Fish body condition among zones.** Relative condition factor (*Kn*) by zone for (A) *Chaetodon capistratus* and (B) *Hypoplectrus puella*. Values of 1.0 (red line) and above represent optimal condition, whereas values below 1.0 indicate suboptimal condition.

**A**


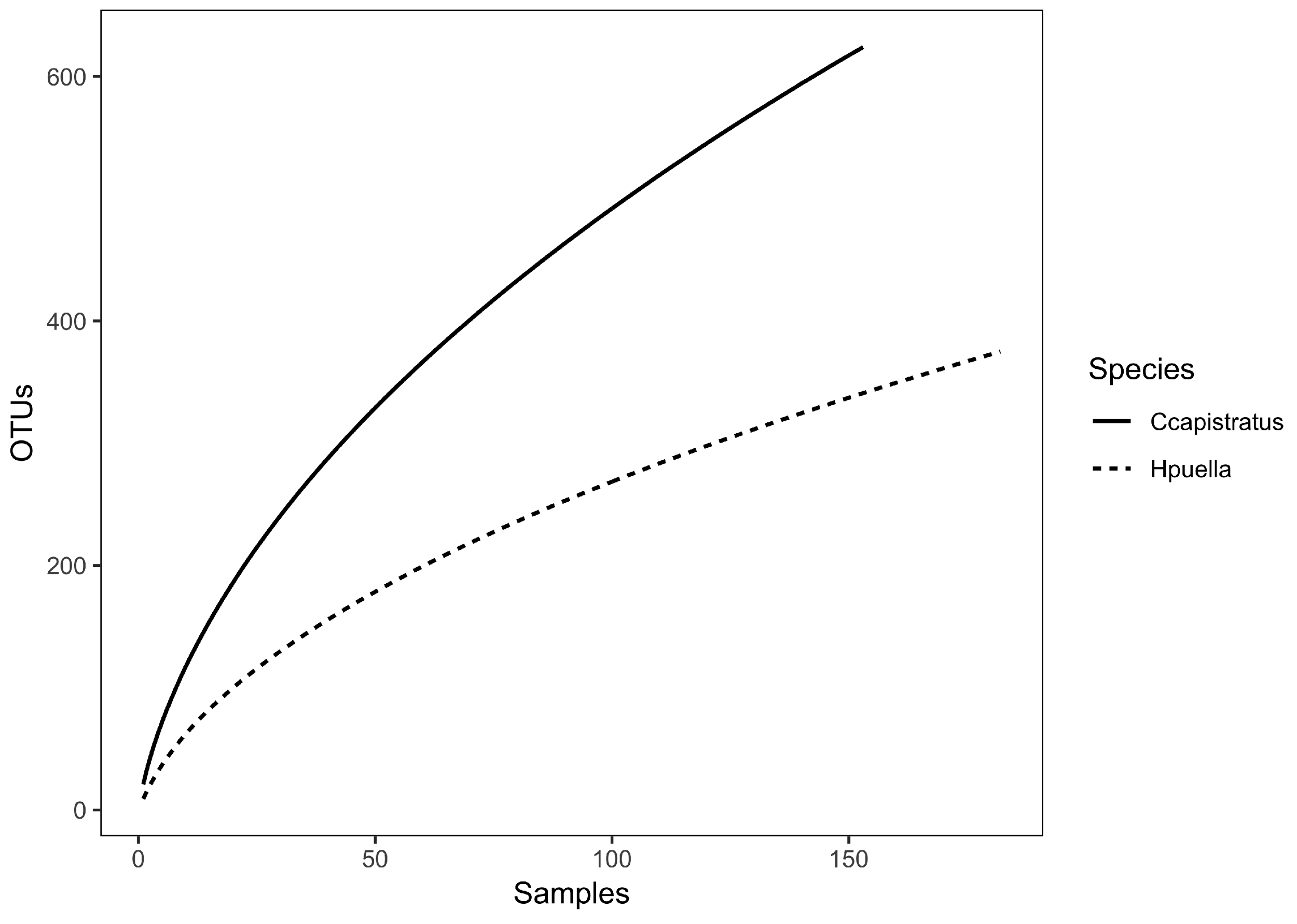


**B**


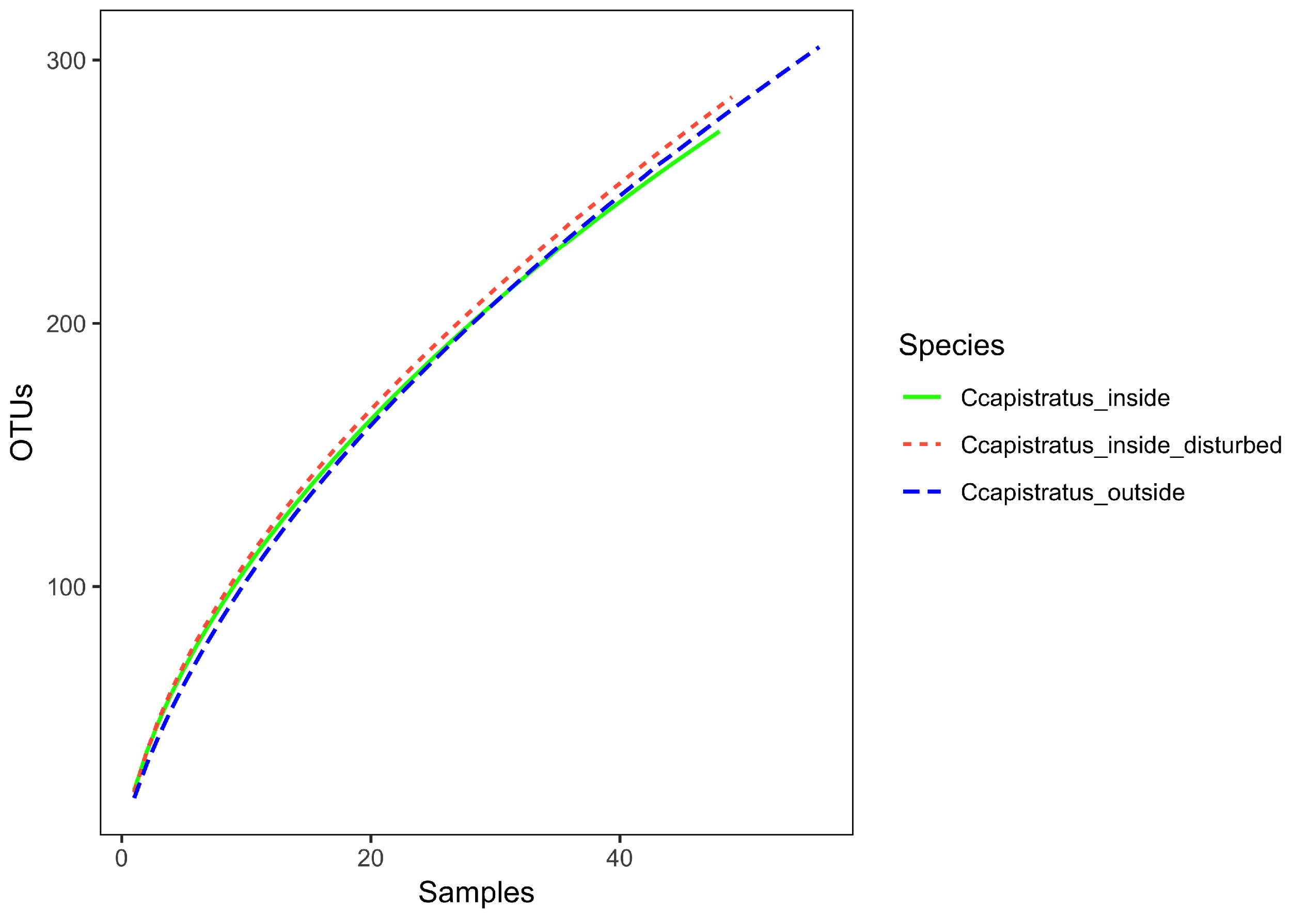


**C**


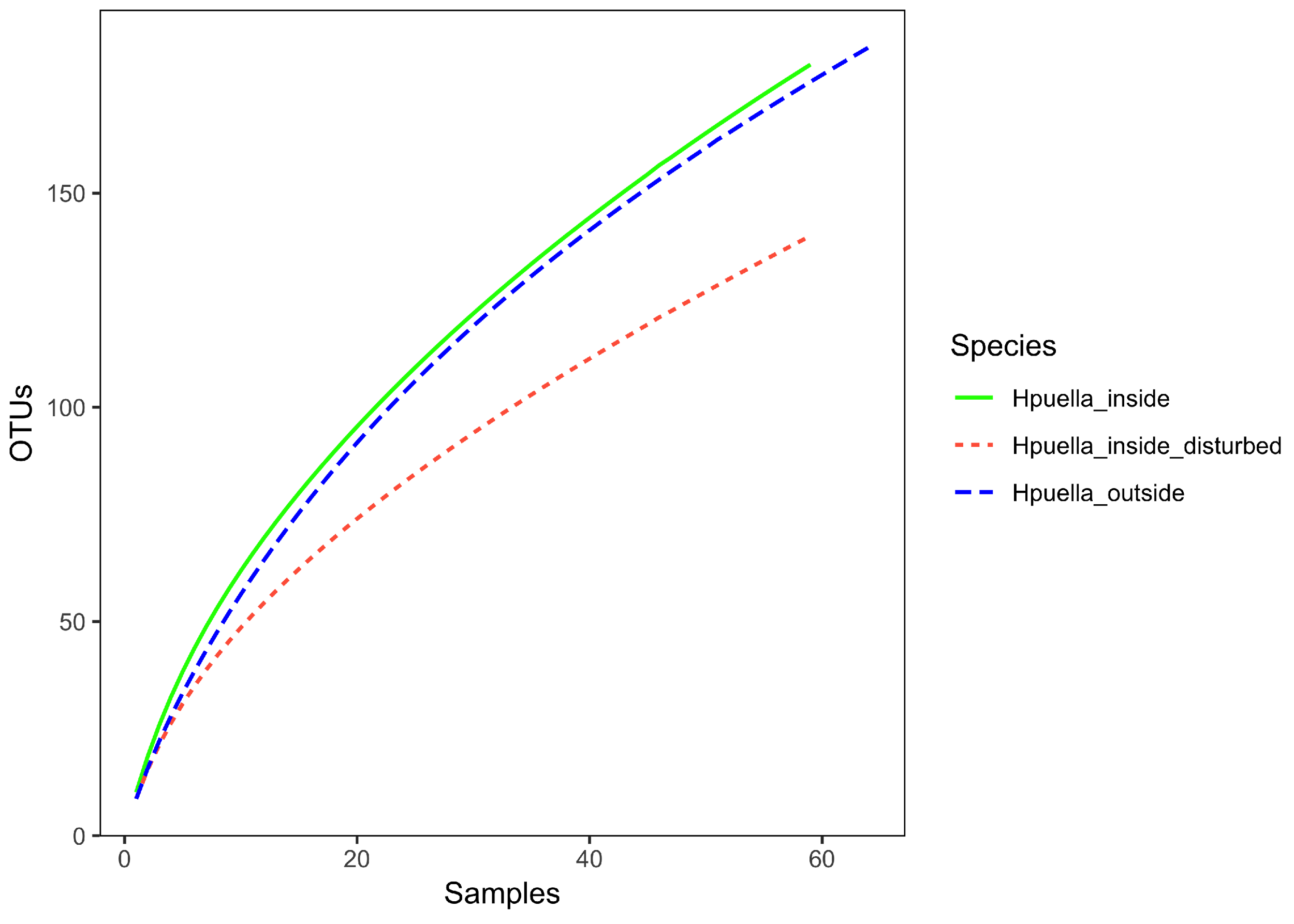


**Figure S8.** **Sample-based rarefaction curves.** (A) for both study species across all samples, (B) *Chaetodon capistratus* and (C) *Hypoplectrus puella* both by reef zone.


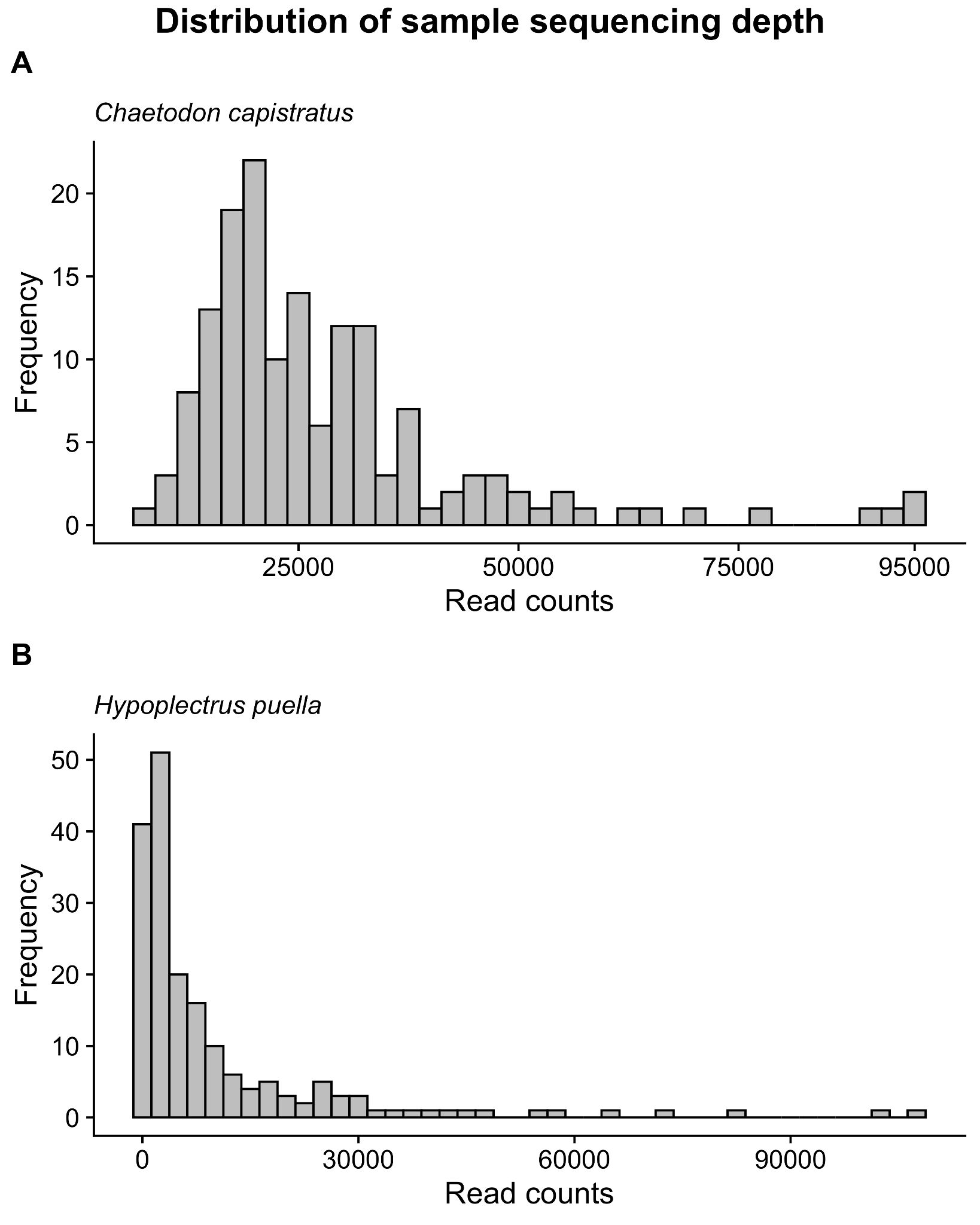


**Figure S9.** **Sequencing depth by samples and species**. The distribution of sequencing depth (sequencing read counts) across (A) stomach content samples of *Chaetodon capistratus* and (B) intestinal content samples of *Hypoplectrus puella* including all sequences delineated as Metazoa (kingdom Animalia).


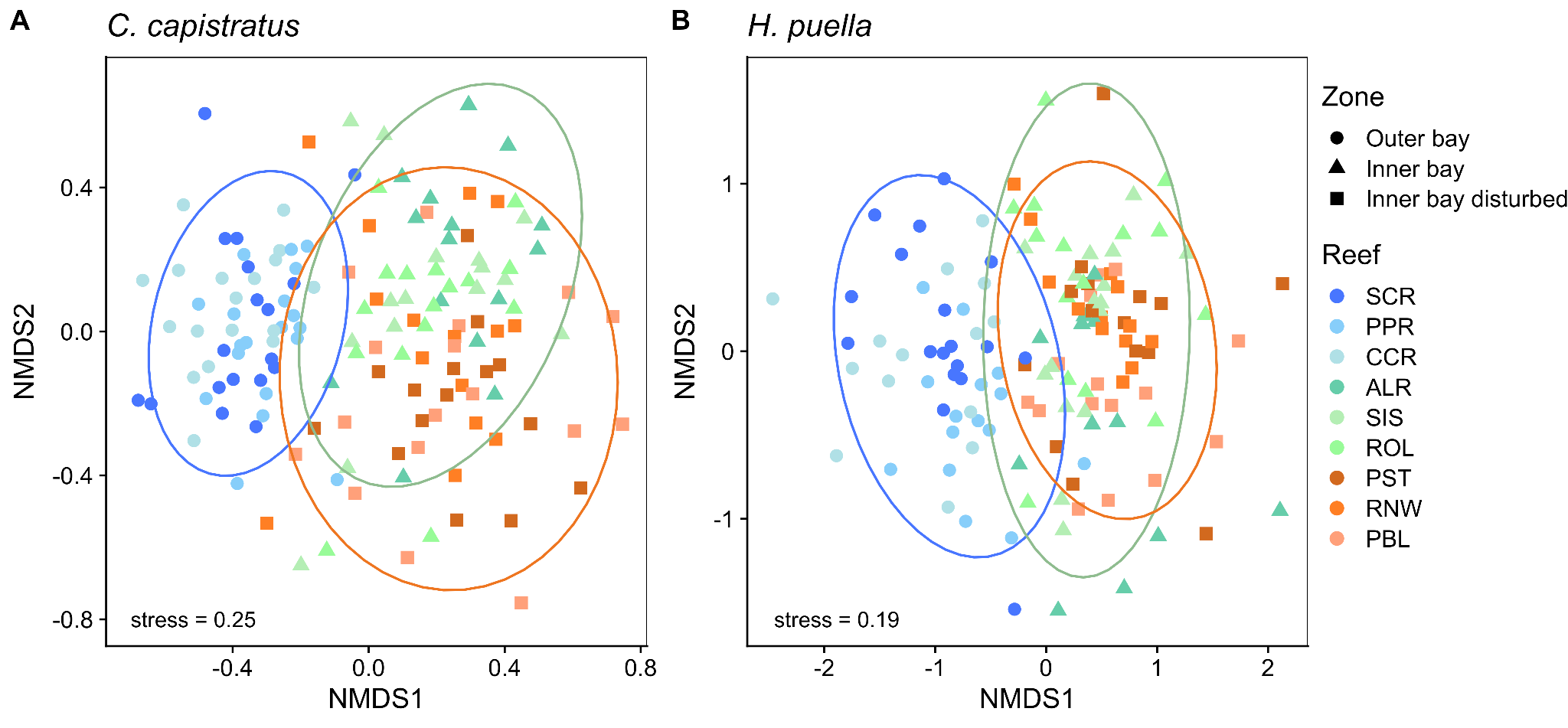


**Figure S10. Differences in fish diet composition based on dietary metabarcoding among zones.** Nonmetric multidimensional scaling (NMDS) plots are based on Jaccard dissimilarity matrices of occurrence data for individuals of (A) *Chaetodon capistratus* and (B) *Hypoplectrus puella*. Dots depict fish individuals; reef zones are coded by shapes and colour: blue = outer bay, green = inner bay, and orange = inner bay disturbed.

**Figure S11. Diet composition of *Chaetodon capistratus*.** (A) depicts all eukaryotes within the phylum Cnidaria found in the diet at the order level, and (B) at the family level (or alternatively the next higher level that could be taxonomically assigned). The diet changes across reefs from Salt Creek (SCR, highest coral cover, far left) to Punta Puebla (PBL, lowest coral cover, far right).

**Figure S12** **Diet composition of *Hypoplectrus puella*.** (A) shows the nine most common prey phyla (or alternatively the next higher level that could be taxonomically assigned (i.e., k = kingdom) across nine study reefs. Arthropods dominate the diet across all reefs and habitat zones despite different levels of coral cover (i.e., from Salt Creek (SCR, highest coral cover, far left) to Punta Puebla (PBL, lowest coral cover, far right). (B): Within only arthropods at the order level (or alternatively the next higher level that could be taxonomically assigned; i.e., c = class, p = phylum), it becomes apparent that more planktonic taxa (i.e., calanoid copepods) are consumed with decreasing coral cover (towards the right) and more decapods (including class Malacostraca) with increasing coral cover (towards the left).


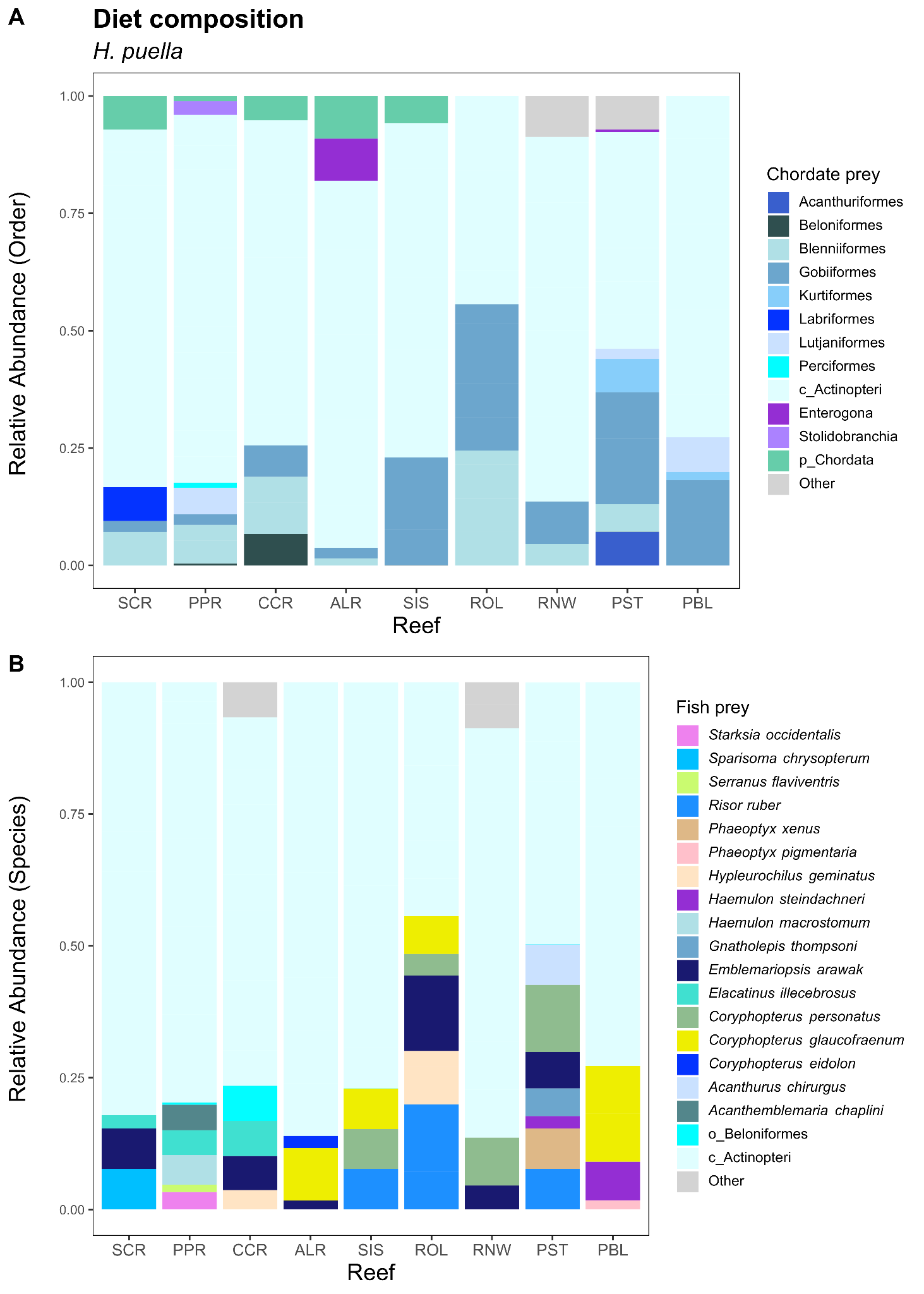


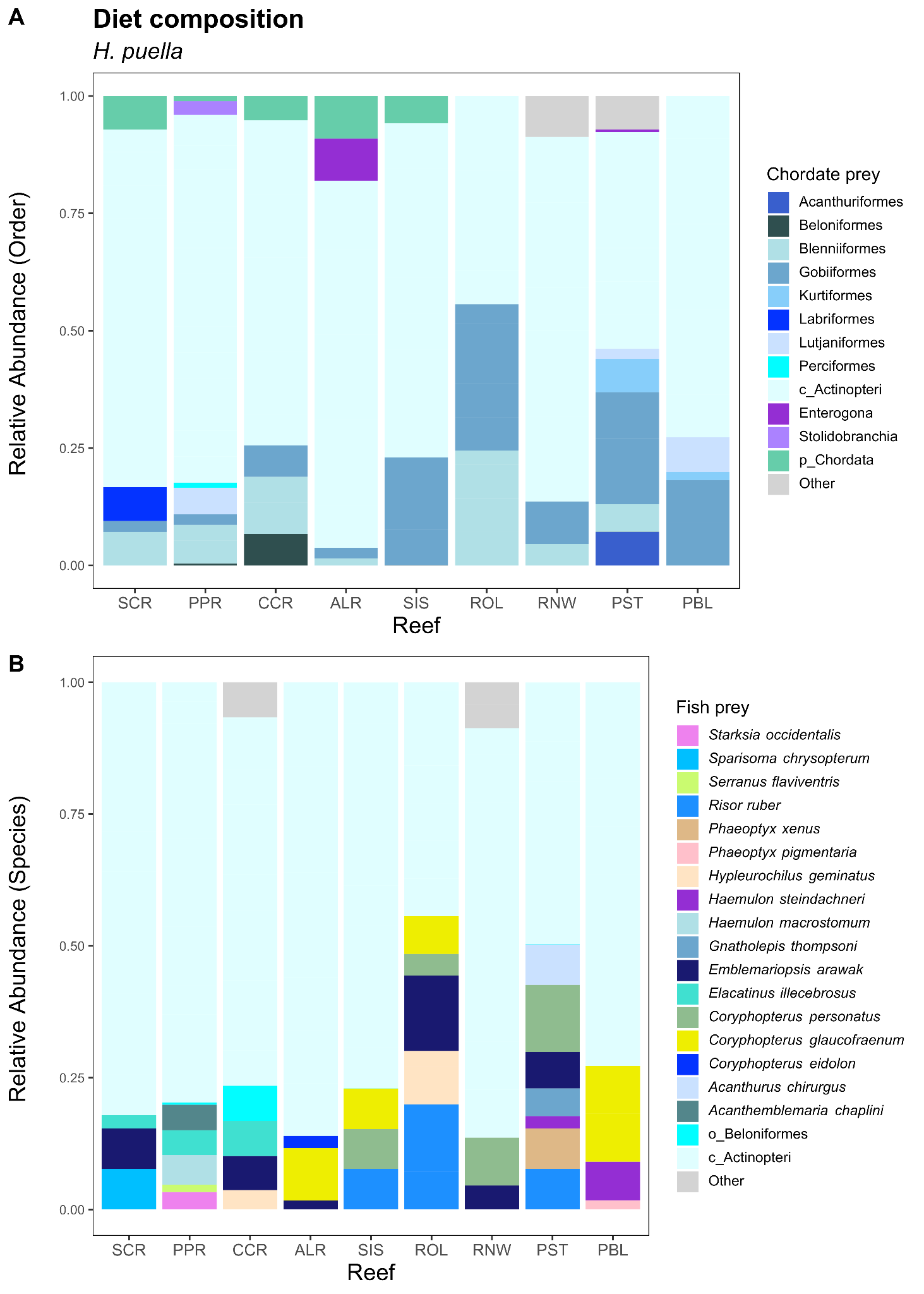


**Figure S13. Diet composition** **of *Hypoplectrus puella* (fish prey).** (A) Composition within phylum Chordata by order (or alternatively the next higher level that could be taxonomically assigned; c = class; p = phylum), and (B) including only fish prey at species level (or alternatively the next higher level that could be taxonomically assigned; o = order; c = class).

**Supplementary Tables**

**Table S1.** The versatile COI primer pair (Geller et al., 2013; Leray et al., 2013) that was used in this study.

| **Primer_name** | **Primer_sequence (5'-3')** |
| --- | --- |
| mlCOIintF | GGWACWGGWTGAACWGTWTAYCCYCC |
| jgHCO2198 | TAIACYTCIGGRTGICCRAARAAYCA |

**Table S2.** Species-specific blocking primer sequences for two coral reef fishes, *Hypoplectrus puella* and *Chaetodon capistratus.*

| **Primer_name** | **Primer_sequence (5'-3')** |
| --- | --- |
| Hpuella-Blocker | CAAAGAATCAGAATAGATGTTGGTAAAGA-C3 |
| Ccapistratus-Blocker | CAAAGAATCAGAACAGGTGTTGGTAAAGA-C3 |

**Table S3. Differences in invertebrate densities.** One-way Analysis of Variance (ANOVA) results testing whether the mean densities of invertebrates overall, specific groups and taxa differed significantly among three reef zones comprising two (outer bay) or three (inner bay, inner bay disturbed) reefs (n = 8). Invertebrates (> 2 mm) were collected from dead coral habitat using three quadrates (50 x 50 cm) per reef. Significant differences are depicted in bold.

**
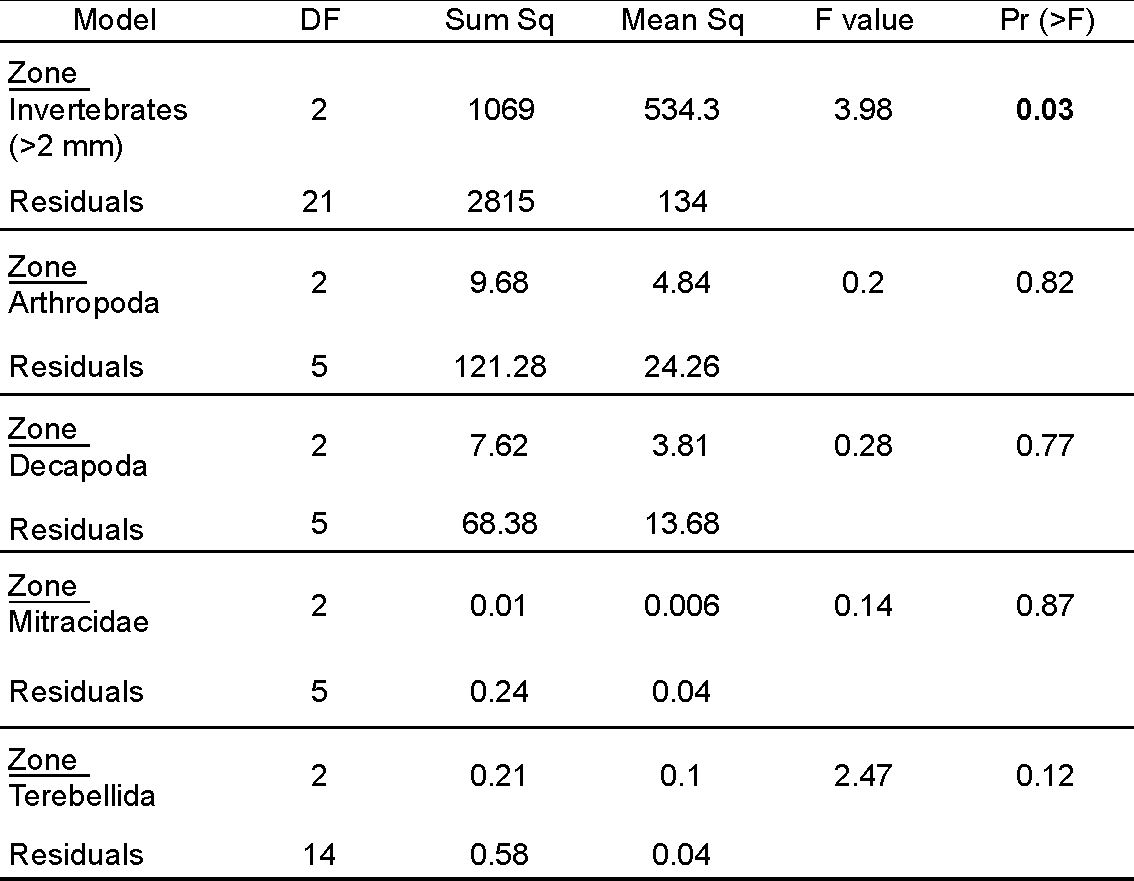
**

**Table S4. Pairwise comparisons of invertebrate mean densities.** Post hoc Tukey test results showing which pairwise comparisons of invertebrate mean densities are responsible for significant differences among three zones as detected by ANOVA (Table S3). Invertebrate mean densities significantly differed between the outer bay zone and inner bay disturbed zone (depicted in bold).

**Table S5**. **Fish length and weight between zones.** (A) Mean (±SD) total length (mm) and wet weight (g) of fish by reef zone, followed by (B) pairwise post hoc comparisons among zones for *Chaetodon capistratus* and *Hypoplectrus puella*. Dunn’s test was used for nonparametric data and Tukey’s HSD for parametric data. Test statistics are Z-values (Dunn’s test) and t-values (Tukey’s HSD).

**A**

**B**

**Table S6**. **Comparison of growth predictions.** Level of significance for differences in Von Bertalanffy Growth Function (VBGF) parameters between zones based on bootstrapped confidence intervals for *Chaetodon capistratus* (N = 158) and *Hypoplectrus puella* (N = 127). Linf is the asymptotic length or length infinitive, K the growth rate at which Linf is approached, and t0 the (hypothetical) point in time at which an individual is of length zero.


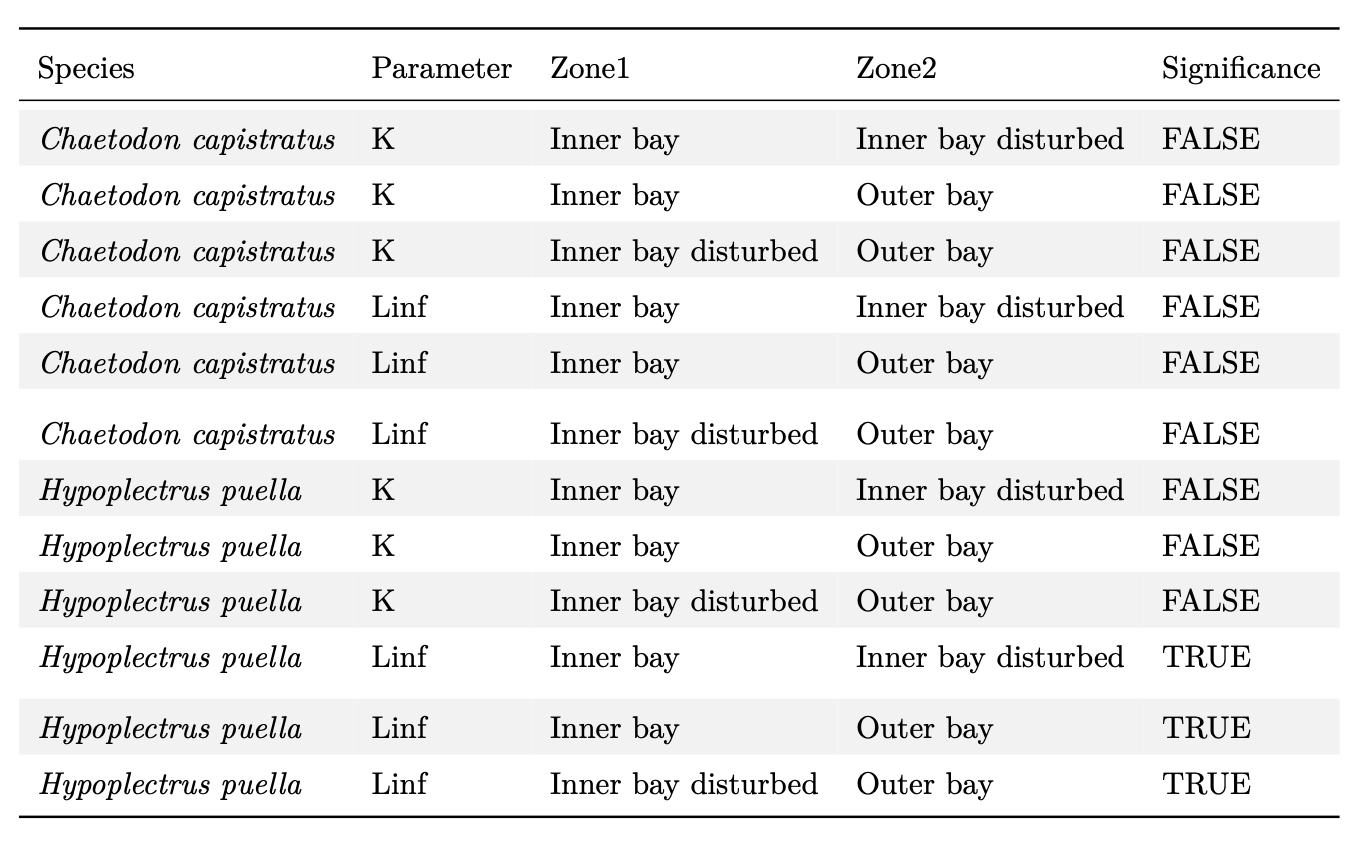


**Table S7. Fish diet composition among zones.** PERMANOVA results of differences in diet composition among three habitat zones considering read abundance (Bray Curtis dissimilarity) and occurrence data (Jaccard Index).

**Table S8. Pairwise comparisons of fish diet composition.** Pairwise PERMANOVA results of differences in diet composition considering read abundance (Bray Curtis dissimilarity) and occurrence data (Jaccard Index).


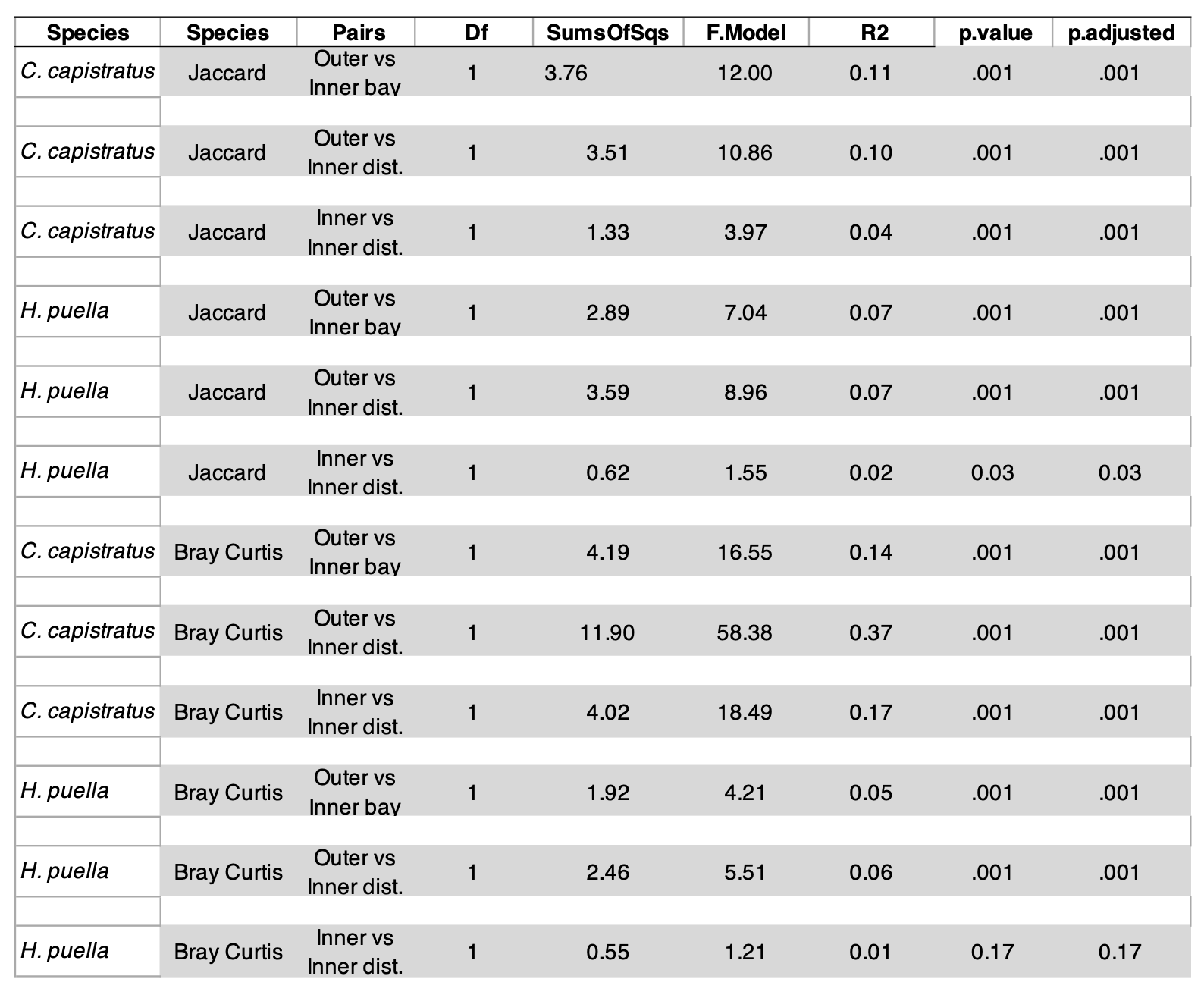


**Supplementary Methods**

**I. Fish collection**To avoid cross-contamination between fish samples*,* we strictly followed the following procedures during fish sample preparation and DNA extraction: First, sterile gloves were changed. Second, the flow-hood was DNA de-contaminated using 10% Sodium Hypochlorite followed by a 90% EtOH rinse. Third, scissors and forceps were placed in a 10% Sodium Hypochlorite bath for 15 min immediately after use, thoroughly rinsed with Milli-Q water and subsequently 70% ethanol and flamed to remove any remaining bleach, water contaminants or tissue.

**III.** **DNA Extraction**Between 0.05 and 0.25 g of prey tissue per sample from *C. capistratus*’ stomach contents and between 0.05 and 0.25 g of *H. puella* digesta (intestinal contents) were added to individual Eppendorf tubes containing power beads with bead solution, C1 solution (60 ul) and 20 ul proteinase K (0.4 mg.mL-1) to enhance the lysis of animal tissue as well as algae. Because yield was low in an initial set of five extractions of intestinal content samples, we briefly vortexed the Eppendorf tubes with digesta before an additional incubation step of 15 min at 60°*C* with 1000 rpm agitation to allow for beginning of tissue lysis by Proteinase K before mechanical disruption by vortexing with beads. Samples were then vortexed for 5 min on the Vortex Genie 2 with vortex adapter followed by a 105 min incubation at 60°*C* with 1000 rpm agitation. The Eppendorf tubes containing stomach content samples were vortexed for 5 min using a Vortex Genie 2 (Scientific Industries) with vortex adapter and subsequently incubated at 60°*C* for two hours with agitation (1000 rpm) on an Eppendorf™ Thermomixer™ R. The longer incubation step recommended in previous studies (Leray et al., 2015; Wangensteen and Turon, 2017) helped lyse hard-shelled invertebrates as well as coral and potentially algae. DNA extracts were diluted 5 times in nuclease-free water and the molecular weight of the extracted genomic DNA was assessed with electrophoresis of GelRed™-stained DNA on a 1.5% agarose gel. DNA concentration (ng/ul) was quantified with a Quant-iT™ dsDNA High-Sensitivity Assay Kit using an Invitrogen Qubit® Fluorometer (Life Technologies), before storing DNA extracts at *−*20°*C*.

**III.** **Metabarcoding library preparation**
We followed a previously published protocol for sample multiplexing that uses a combination of tailed PCR primers and ligation of indexed adapters (Leray et al., 2016). To do so we used matching oligonucleotide indices (Binladen et al., 2007; Coissac et al., 2012) on both forward and reverse primers to prevent tag-jumping—a process that may generate spurious assignments of sequence reads to samples (Schnell et a., 2015). A total volume of 20 ul was used in each PCR reaction comprised of 2 x PCR buffer (Clonetech) with 1.8 mM MgCl2, 3% DMSO, 0.2 mM dNTP, 0.4 Advantage TAQ polymerase (Clonetech), 1 M of forward and reverse primer respectively (mlCOIintF and jgHCO2198) (Leray et al., 2013; Geller et al. 2013) and 1 ng/ul of DNA template. A PCR blank (using 1 ng/l nuclease free water instead of DNA template) was included in each PCR replicate run, and positive controls were included in PCRs of gut samples. Each PCR thermal cycle consisted of an initial denaturation step of 5 minutes at 95°*C* followed by 38 cycles of 95°*C* (30 seconds), 48°*C* (30 seconds), 72°*C* (45 seconds), with a final 5 minute extension at 72°*C* and a final cooling step of 4°*C*. PCRs were assessed with electrophoresis on 1.5% agarose gel stained with GelRed™. The three PCR replicates generated for each sample were pooled. PCR product clean-ups were performed using DNA Purification Solid Phase Reversible Immobilization (SPRI) magnetic beads (KAPA Pure Beads, Roche) using a bead:DNA ratio of 1.6:1. To achieve similar numbers of reads per sample after sequencing, cleaned-up amplicon DNA was normalized at 5ng/ul using nuclease-free water. Equimolar amplicon DNA of samples with each unique tag were then pooled into the respective adapter groups for adapter ligation. The TruSeq DNA PCR-free LT library Prep Kit (Illumina) was used for library preparation following the manufacturer's protocol.

**References**

Binladen, J., Gilbert, M. T. P., Bollback, J. P., Panitz, F., Bendixen, C., Nielsen, R., & Willerslev, E. (2007). The use of coded PCR primers enables high-throughput sequencing of multiple homolog amplification products by 454 parallel sequencing. *PLoS ONE*, *2*(2). https://doi.org/10.1371/journal.pone.0000197

Coissac, E., Riaz, T., & Puillandre, N. (2012). Bioinformatic challenges for DNA metabarcoding of plants and animals. *Molecular Ecology*, *21*(8), 1834–1847. https://doi.org/10.1111/j.1365-294X.2012.05550.x

Geller, J., Meyer, C., Parker, M., & Hawk, H. (2013). Redesign of PCR primers for mitochondrial cytochrome c oxidase subunit I for marine invertebrates and application in all-taxa biotic surveys. *Mol Ecol Resour*, *13*(5), 851–861. https://doi.org/10.1111/1755-0998.12138

Leray, M., Haenel, Q., & Bourlat, S. J. (2016). Preparation of amplicon libraries for metabarcoding of marine eukaryotes using illumina MiSeq: The adapter ligation method. In *Methods in Molecular Biology* (Vol. 1452, pp. 209–218). Humana Press Inc.

Leray, M., Meyer, C. P., & Mills, S. C. (2015). Metabarcoding dietary analysis of coral dwelling predatory fish demonstrates the minor contribution of coral mutualists to their highly partitioned, generalist diet. *PeerJ*, *3*, e1047–e1047. https://doi.org/10.7717/peerj.1047

Leray, M., Yang, J. Y., Meyer, C. P., Mills, S. C., Agudelo, N., Ranwez, V., Boehm, J. T., & Machida, R. J. (2013). A new versatile primer set targeting a short fragment of the mitochondrial COI region for metabarcoding metazoan diversity: Application for characterizing coral reef fish gut contents. *Frontiers in Zoology*, *10*(1), 34–34.

Schnell, I. B., Bohmann, K., & Gilbert, M. T. P. (2015). Tag jumps illuminated—Reducing sequence-to-sample misidentifications in metabarcoding studies. *Mol Ecol Resour*, *15*(6), 1289–1303. https://doi.org/10.1111/1755-0998.12402

Wangensteen, O. S., & Turon, X. (2017). Metabarcoding techniques for assessing biodiversity of marine animal forests. In *Marine Animal Forests: The Ecology of Benthic Biodiversity Hotspots* (pp. 445–473). Springer International Publishing.
